# Potential impact of pre-exposure prophylaxis for female sex workers and men who have sex with men in Bangalore, India: a mathematical modelling study

**DOI:** 10.7448/IAS.19.1.20942

**Published:** 2016-09-07

**Authors:** Kate M Mitchell, Holly J Prudden, Reynold Washington, Shajy Isac, Subramanian P Rajaram, Anna M Foss, Fern Terris-Prestholt, Marie-Claude Boily, Peter Vickerman

**Affiliations:** 1 Social and Mathematical Epidemiology Group (SaME), London School of Hygiene and Tropical Medicine, London, UK; 2 Department of Infectious Disease Epidemiology, Imperial College London, London, UK; 3 Karnataka Health Promotion Trust, Bangalore, India; 4 St John's Research Institute, Bangalore, India; 5 Center for Global Public Health (CGPH), University of Manitoba, Winnipeg, Canada; 6 CHARME-India Project, Bangalore, India; 7 School of Social and Community Medicine, University of Bristol, Bristol, UK

**Keywords:** key population, high-risk group, prevention, targeting, prioritizing, focussed intervention, oral PrEP

## Abstract

**Introduction:**

In Bangalore, new HIV infections of female sex workers and men who have sex with men continue to occur, despite high condom use. Pre-exposure prophylaxis (PrEP) has high anti-HIV efficacy for men who have sex with men. PrEP demonstration projects are underway amongst Indian female sex workers. We estimated the impact and efficiency of prioritizing PrEP to female sex workers and/or men who have sex with men in Bangalore.

**Methods:**

A mathematical model of HIV transmission and treatment for female sex workers, clients, men who have sex with men and low-risk groups was parameterized and fitted to Bangalore data. The proportion of transmission attributable (population attributable fraction) to commercial sex and sex between men was calculated. PrEP impact (infections averted, life-years gained) and efficiency (life-years gained/infections averted per 100 person-years on PrEP) were estimated for different levels of PrEP adherence, coverage and prioritization strategies (female sex workers, high-risk men who have sex with men, both female sex workers and high-risk men who have sex with men, or female sex workers with lower condom use), under current conditions and in a scenario with lower baseline condom use amongst key populations.

**Results:**

Population attributable fractions for commercial sex and sex between men have declined over time, and they are predicted to account for 19% of all new infections between 2016 and 2025. PrEP could prevent a substantial proportion of infections amongst female sex workers and men who have sex with men in this setting (23%/27% over 5/10 years, with 60% coverage and 50% adherence), which could avert 2.9%/4.3% of infections over 5/10 years in the whole Bangalore population. Impact and efficiency in the whole population was greater if female sex workers were prioritized. Efficiency increased, but impact decreased, if only female sex workers with lower condom use were given PrEP. Greater impact and efficiency was predicted for the scenario with lower condom use.

**Conclusions:**

PrEP could be beneficial for female sex workers and men who have sex with men in Bangalore, and give some benefits in the general population, especially in similar settings with lower condom use levels.

## Introduction

India has a concentrated HIV epidemic. In Bangalore, as elsewhere in southern India, the highest HIV levels are found amongst female sex workers (FSWs; 8.0% prevalence 2009), FSWs’ commercial clients (2.4%, 2007), and men who have sex with men (MSM; 16.5%, 2009) [1–4]. HIV prevalence among the general population is much lower (0.75% amongst women attending antenatal care (ANC) in 2012) [5]. Previous modelling suggests that most HIV transmission in southern India is driven by commercial sex [6].

Following the *Avahan* intervention, which promoted condom use for FSWs and MSM [7], reported condom use increased. In 2006, 74% of MSM reported using condoms consistently with casual male sex partners, and in 2011, 90% of FSWs reported using condoms consistently with new commercial clients [4,8]. However, FSWs report much lower condom use with non-commercial partners [9].

Between surveys conducted in 2006 and 2009, HIV prevalence amongst FSWs in Bangalore fell, consistent with reported increases in condom use [7], but remained level between 2009 and 2011 [10]. HIV prevalence amongst MSM remained similar between 2006 and 2009 [11].

Oral daily pre-exposure prophylaxis (PrEP), which involves HIV-negative individuals taking a daily tablet containing tenofovir or tenofovir/emtricitabine, has been shown to reduce the risk of HIV acquisition for MSM [12–14] and heterosexuals, provided medication is taken regularly [15–18]. The iPrEx trial found an overall reduction in HIV acquisition amongst MSM of 44% in the entire cohort, rising to 92% amongst those with detectable drug in their blood [12], while the IPERGAY and PROUD trials both reported an overall 86% reduction in HIV acquisition amongst MSM [13,14]. Trials amongst heterosexuals found HIV risk reductions between 0 and 75%, with efficacy strongly related to PrEP adherence [19]; in the Partners PrEP study, an efficacy of 90% for tenofovir/emtricitabine was estimated amongst those with detectable drug in their blood [15].

While reported condom use by FSWs and MSM is high, new infections continue to occur in many Indian settings, and there is interest in finding additional interventions which might reduce HIV incidence amongst these populations to low levels. PrEP is a promising candidate which, unlike condoms, can be used without a partner's knowledge. A PrEP demonstration project is underway amongst FSWs in two Indian cities, Mysore and Kolkata [20]. Because of this, and the demonstrated efficacy of PrEP for MSM, the potential impact of PrEP for these groups in India is of great interest. Here, we used mathematical modelling to estimate the contribution made by these key populations to the HIV epidemic in Bangalore, the impact of offering PrEP to FSWs and/or MSM in Bangalore, and the population-level impact and efficiency of different PrEP prioritization strategies.

## Methods

### Model

We extended a deterministic, compartmental model of HIV transmission amongst MSM in Bangalore [21] to include FSWs, FSW clients, former MSM, FSWs and clients, and low-risk males and females. Individuals enter the model on sexual debut. The model divides HIV-negatives by PrEP status, and HIV-positives by infection stage (acute, CD4 >500, 350 to 500, 200 to 350, <200 cells/µl, pre-AIDS, AIDS) and engagement with HIV care and treatment (never testing/testing but not in care/in pre-anti-retroviral therapy (ART) care/on ART/no longer on ART). ART is initiated following testing and pre-ART care, or after becoming symptomatic (CD4 < 200 cells/µl).

MSM are divided into three groups, related to the role taken in sex with men: *kothi* and *hijra* (usually receptive), *panthi* and bisexual (usually insertive) and double deckers (both insertive and receptive). *Kothi/hijra* and double deckers are collectively called “high-risk” MSM (HR-MSM).

FSWs are divided by reported condom use with new clients (“always” (high-condom) versus “often/sometimes/never” (low-condom)), with women recruited into the high-condom group over time reflecting historical changes in condom use.

Individuals leave the population due to natural or AIDS-related mortality, and those in key populations can leave them, entering the former MSM/FSW/client groups. The modelled population grows over time, in line with observed Bangalore population growth.

Those on ART progress through the same HIV infection stages as other HIV-positive people, but more slowly. Temporary cessation of ART, with rapid ART re-initiation, is not explicitly modelled. Long-term dropouts from ART, who are not rapidly re-initiated, are assumed to only seek treatment when they become symptomatic; in our model this is approximated as reaching CD4 < 200 cells/µl.

HIV was seeded in the model amongst MSM, FSWs and clients in 1986 [22] (the earliest year with HIV prevalence data for southern India), and the epidemic was simulated until 2037.

Condom use and rates of HIV testing and linkage into care were assumed to increase linearly over time in line with data, remaining constant after the latest data estimate (Supplementary Figure 2).

See Supplementary File for further model details, schematic (Supplementary Figure 1) and equations.

### Parameterization and fitting

HIV prevalence data and behavioural data used to parameterize the model came from integrated behavioural and biological assessments [23], conducted amongst FSWs, MSM and clients in Bangalore at two or three time points between 2006 and 2012. Additional behavioural data were obtained from special behavioural surveys, conducted amongst FSWs and MSM in Bangalore in 2006 [24]. Condom use trends were estimated using data on current levels and duration of consistent condom use [25] and trends in HIV testing rates were estimated by fitting linear trend-lines to self-reported testing rates in different surveys [4]. Condom use in regular (non-commercial) partnerships was based upon levels reported in such partnerships by FSWs, clients and MSM [4].

Population sizes were estimated from MSM and FSW mapping surveys [26], population-based surveys from neighbouring districts [27], and district census estimates.

Biological parameters and the efficacy of condoms and ART for reducing HIV transmission were obtained from published literature.

Rates of linkage to HIV care, numbers of adults on ART (2007 to 2012) and ART coverage (2007–2009) were estimated from state-level data [28–30].

Selected parameters are given in Table 1, details of all model parameters in Supplementary Table 1 (Supplementary File).

The model was fitted to HIV prevalence and ART coverage in two stages within a Bayesian framework [31] (see Supplementary File).

**Table 1:** Selected sexual behaviour and intervention parameters

Parameter	Value	Range
Sexual behaviour		
Commercial sex acts per year for FSW	384.0	359.0–410.0
Commercial sex acts per year for non-MSM clients	19.6	17.9–21.4
MSM sex partners per year for *kothi/hijra*	130.5	93.0–168.0
MSM sex partners per year for double deckers	69.5	53.0–86.0
MSM sex partners per year for *panthi*/bisexuals	49.0	2.0–96.0
For those with a regular partner, number of vaginal sex acts per year	108.4	90.8–127.6
Condom use		
% of commercial sex acts in which condom used in 2011	90.0	87.3–92.7
% of anal sex acts between MSM in which condom used in 2007	70.5	63.7–77.2
% of sex acts in which condom used in regular partnerships	16.1	5.1–27.0
HIV testing		
% of MSM testing for HIV annually after 2009	58	50–66
% of FSW testing for HIV annually after 2011	90	87–92
% of clients, former MSM, former FSW, low-risk population testing for HIV annually after 2007	11	7–14
PrEP intervention		
Proportion of MSM offered and initiating PrEP upon testing negative for HIV to achieve required PrEP coverage	20%: 0.105/*τ*_*i,Max*_ 60%: 0.59/*τ*_*i,Max*_^a^	fixed
Proportion of FSW offered and initiating PrEP upon testing negative for HIV to achieve required PrEP coverage	20%: 0.17 60%: 1.0	fixed
Rate of PrEP dropout, all groups, per person per year	0.2	fixed
Pre-ART care linkage and dropout		
Proportion linking to pre-ART care, all groups, 2011 onwards	0.8	0.7–0.9
Ratio of dropout from pre-ART care relative ART dropout rate	2	1–3
ART		
Rate at which those in pre-ART care, in HIV stage *i*, initiate ART, per year	Pre-2004, 0 all *i* 2004–2011, 2 if CD4 < 200, 0 otherwise 2011 onwards, 2 if CD4 < 350, 0 otherwise	fixed
Rate of initiating ART per year due to symptoms in AIDS stage	Pre-2004, 0 2004 onwards, 1	0–2
Relative rate of initiating ART due to symptoms in pre-AIDS stage relative to AIDS stage		0.1–1
Relative rate of initiating ART due to symptoms with CD4 < 200 relative to those in pre-AIDS stage		0.1–1
Rate of ART dropout, per year	0.04	0.01–0.07
Factor by which HIV progression rates are multiplied when on vs off ART	1/3	fixed
Intervention efficacies in reducing HIV transmission risk		
Per-sex-act efficacy of ART in anal or vaginal sex (%)	92	26–100
Per-sex-act efficacy of condoms in vaginal sex (%)	80	66–94
Per-sex-act efficacy of condoms in anal sex (%)		61–94
Per-sex-act efficacy of PrEP (%)	93	fixed

For sources and details of all other parameters, see Supplementary Table 1.

a *τ*_*i,Max*_=% of MSM testing for HIV annually after 2009.

### Analysis

#### Baseline prevalence and incidence

Overall HIV prevalence was compared with Bangalore ANC and Mysore data. The time of HIV elimination (<1 new infection/1000 person-years) was estimated.

#### Population attributable fraction

The population attributable fraction (PAF) quantifies the contribution of a particular risk factor to cases of disease. We used PAF to understand the factors driving HIV transmission, which is crucial for designing effective prevention interventions. The model was used to estimate the PAF of commercial sex, sex between men and regular partnerships to the overall HIV epidemic, and to the FSW and MSM epidemics, over sequential 10-year periods from 1986. The PAF was estimated by determining the relative reduction in the number of HIV infections over each 10 year period if the transmission probability for commercial sex, sex between men or regular partnerships was set to 0 over that period.

#### PrEP intervention scenarios

PrEP interventions were modelled commencing January 2017. HIV-negative individuals were recruited onto PrEP at a constant rate, and stopped taking PrEP if they acquired HIV infection or dropped out (assumed 20% annually). PrEP was offered only to current MSM or FSW, those leaving these groups were assumed to stop taking PrEP. Drug resistance was not included. PrEP efficacy was assumed to be 93% under perfect adherence (between the efficacy estimates for daily dosing from the iPrEx and Partners PrEP trials [32,33]). PrEP coverage for prioritized groups was calibrated to reach 20 or 60% coverage of HIV-negatives after five years. Different adherence scenarios were explored: 30, 50 or 75% adherence, as found in different trials [12,18,33]. Overall PrEP effectiveness was calculated as the product of the assumed efficacy and adherence; this relationship approximates that seen in the iPrEx and Partners PrEP trials, with adherence measured by detectable drug in blood [34] (effectiveness=23%, 47%, 70% for 30%, 50%, 75% adherence scenarios, respectively). Four prioritization scenarios were examined, with PrEP prioritized to all FSWs; HR-MSM; FSWs and HR-MSM; or only low-condom FSWs.

We assessed the effect of a hypothetical change in ART eligibility to all HIV-positives (regardless of CD4 count) starting in 2017.

The effect of imperfect prioritization of PrEP for FSWs was investigated by comparing scenarios where the same amount of PrEP (sufficient for 60% coverage of low-condom FSWs) was distributed amongst low-condom FSWs, all FSWs or high-condom FSWs.

We assessed the possible impact of condom migration by comparing the impact of PrEP when condom use is reduced amongst those using PrEP with the situation where condom use is maintained.

Finally, we examined a scenario with lower condom use amongst key populations to give insights into the possible impact of PrEP in other lower condom use settings. In this second scenario, condom use by FSW and MSM remained constant after 2003 (Supplementary Figures 2 and 3).

#### Outcome measures

Impact within the prioritized groups and in the whole Bangalore population was measured as cumulative number and percentage of infections averted (IA) over five or ten years and life-years gained (LYG) over 20 years. The longer time-frame for LYG is used because of the delay in impact on mortality following the prevention of an infection. Efficiency was defined as LYG or IA per 100 person-years on PrEP. Impacts were not discounted over time.

## Results

### Baseline prevalence and incidence trends

Of one million parameter sets, 115 fitted HIV prevalence and ART coverage data. Most fits suggested declining FSW and client HIV prevalence, in line with data (Supplementary Figure 4a and b), but predicted more rapid HIV prevalence decline amongst MSM than data suggests (Supplementary Figure 4c–e). Although the model was not fitted to overall population HIV prevalence (unavailable for Bangalore), prevalence trends were similar to data for nearby Mysore [35], and female prevalence was similar, although sometimes higher, than Bangalore ANC data [36] (Supplementary Figure 4f–h).

For most model fits (72%), overall HIV incidence was predicted to decline below 1 infection/1000 person-years by 2037 without PrEP, with 50% reaching this threshold by 2015, but 19% of fits predicted that overall incidence would be rising in 2037.

### Population attributable fractions

Between 1986 and 1995, a median 68% of new HIV infections (95% credible interval (CrI) 55 to 84%) were estimated to be attributable to commercial sex, and 27% (13 to 54%) to sex between men, together accounting for 85% (72 to 95%) of new infections (Supplementary Figure 5a). These PAFs declined over time, as condom use amongst key populations and HIV prevalence in the rest of the population increased; we predicted that between 2016 and 2025, 19% (12 to 20%) of new infections would be attributable to commercial sex or MSM. The PAF for regular partnerships involving current MSM, FSWs and clients declined more slowly, from 51% (1986 to 1995) to 38% (2016 to 2025; Supplementary Figure 5b), while the PAF for regular partnerships involving former MSM, FSWs and clients increased from 17 to 60%. Few (<10%) new infections were attributable to partnerships between low-risk men and women. FSWs and (particularly) MSM are estimated to have had a modest effect upon each other's HIV epidemics, and are predicted to account for less than 10% of each other's new HIV infections between 2016 and 2025 (Supplementary Figure 5c).

In Scenario 2, where condom use plateaued after 2003, higher PAFs for commercial sex and sex between men were predicted post-2003, with 36% of new infections between 2016 and 2025 being attributable to either commercial sex or sex between men (Supplementary Figure 5d), 49% to regular partnerships involving former MSM, FSWs or clients (Supplementary Figure 5e), and 19% of new MSM infections attributable to FSW commercial sex (Supplementary Figure 5f).

### PrEP impact: key populations

PrEP coverage time-trends are shown in Supplementary Figure 6.

**Figure 1:**
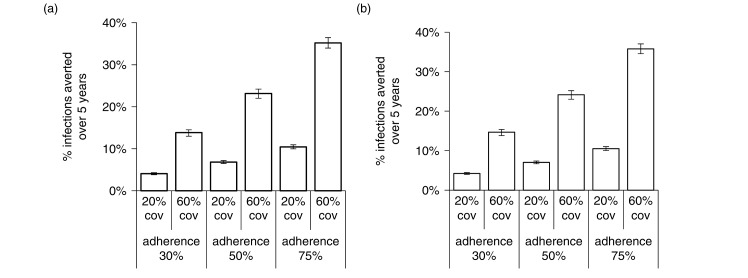
Percentage of infections averted amongst FSW and HR-MSM for PrEP interventions prioritizing each population. Percentage of infections averted amongst (a) all FSWs and (b) HR-MSM over five years, for PrEP interventions prioritizing this population, with PrEP adherence and coverage shown. Bar shows median and error bars are 95% credible interval across 115 parameter sets. Credible intervals give the 2.5th and 97.5th percentiles of estimates across all parameter combinations.

A PrEP intervention prioritized to and reaching 60% of all HIV-negative FSWs after five years, with 50% PrEP adherence (47% effectiveness), averted 23% of FSWs infections (22 to 24%) over five years (Figure 1a). Similar results were seen prioritizing HR-MSM (Figure 1b). Five-year impact varied linearly with assumed adherence (Figure 1a and b) and coverage.

Impact increased slightly over time, with 26%/29% of FSW/HR-MSM infections being prevented after 10 years for 60% coverage and 50% adherence (data not shown).

PrEP gave sustained incidence reductions among FSWs and MSM – after five years, an intervention reaching 60% of HR-MSM and FSWs reduced incidence amongst FSWs by 19, 31 or 45% with 30, 50 or 75% adherence, respectively, with similar reductions among HR-MSM (Supplementary Figure 7).

**Figure 2:**
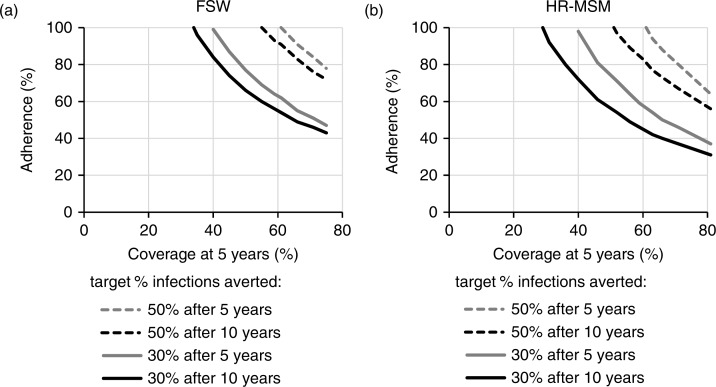
Coverage and adherence required to meet impact targets. Combinations of coverage (after five years) and adherence required to meet impact targets of 30 or 50% of infections averted over five or ten years amongst (a) FSW and (b) HR-MSM, when that group is prioritized with a PrEP intervention. Results are shown for the best-fit parameter set.

Figure 2 show the combinations of adherence and coverage required to meet different impact targets. Reaching 60% of FSWs, 56 or 93% adherence was required to prevent 30 or 50% of FSW infections after 10 years, respectively (Figure 2a). To reach these targets after only five years, higher adherence (64%) was required to prevent 30% of FSWs infections with 60% coverage, and coverage had to exceed 61% to prevent 50% of FSW infections. Slightly lower coverage and adherence levels were required to reach these targets for HR-MSM (Figure 2b).

### PrEP impact: overall Bangalore population

**Figure 3:**
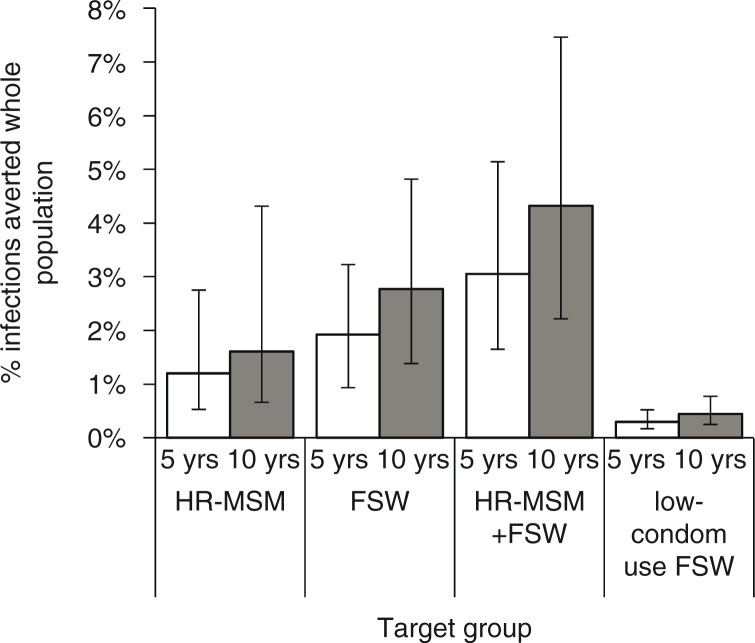
Percentage of infections averted in the whole Bangalore population. Percentage of infections averted in the whole Bangalore population over five and ten years, for an intervention with 50% adherence and 60% coverage of the priority group (as shown). Bar shows median and error bars are 95% credible interval across 115 parameter sets. Credible intervals give the 2.5th and 97.5th percentiles of estimates across all parameter combinations.

The proportion of IA in the whole Bangalore population after five years was low, but greater with FSW prioritization (1.8% (0.9–3.1%), for 50% adherence and 60% coverage) than HR-MSM prioritization (1.2% (0.5–2.9%)). The proportion of IA when both groups were prioritized (2.9%) was almost additive (Figure 3). Impact increased by 50% after 10 years (4.3% prioritizing FSW + HR-MSM). Prioritizing only lower-condom FSWs gave substantially lower population-level impact (Figure 3). With FSW prioritization, large numbers of infections were prevented amongst FSWs, clients and other groups, whereas most infections were prevented amongst MSM with HR-MSM prioritization (Supplementary Figure 8).

HIV elimination (incidence ≤1 infection/1000 person-years) was slightly more likely to occur with PrEP interventions in place, with elimination by 2037 for 79% of model fits, and only 15% of model fits predicting increasing incidence in 2037.

### PrEP efficiency

**Figure 4:**
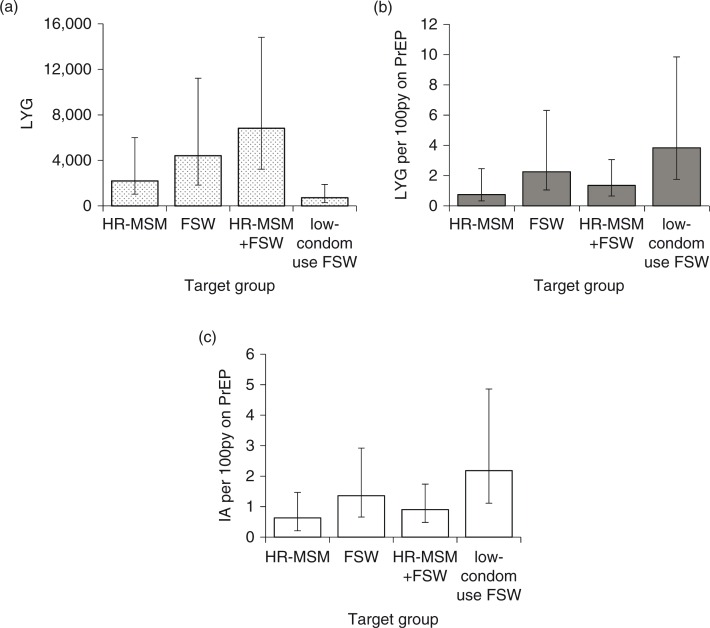
PrEP impact and efficiency in the whole population for different prioritization strategies. (a) Life-years gained, (b) Life-years gained per 100 person-years of PrEP, measured in the whole population over 20 years, and (c) infections averted per 100 person-years of PrEP over five years, when a PrEP intervention with 60% coverage and 50% adherence is prioritized to the different populations shown. Bar shows median and error bars are 95% credible interval across 115 parameter sets.

More life-years were gained in the whole population with FSW compared with HR-MSM PrEP prioritization (Figure 4a). PrEP efficiency was greatest when low-condom FSWs were prioritized (3.7 LYG/100py PrEP after 20 years), followed by all FSWs (2.2 LYG/100py PrEP), with lower efficiency when HR-MSM were prioritized (0.7 LYG/100py PrEP; Figure 4b). Similar patterns were seen with IA: efficiency was 2.2, 1.4 and 0.6 IA/100py PrEP over five years prioritizing low-condom FSWs, all FSWs or HR-MSM (Figure 4c).

### Changes to ART eligibility

PrEP impact among the prioritized group was not affected by ART eligibility changes; impact and efficiency in the whole population declined slightly with expanded ART eligibility from 2017 (Supplementary Figure 9).

### Imperfect prioritization and condom migration

**Figure 5:**
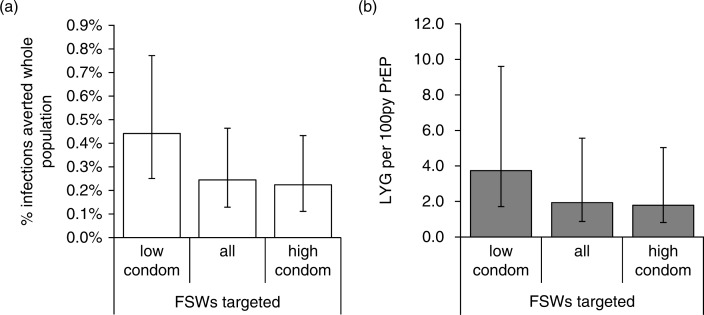
PrEP impact and efficiency in the whole population for different FSW prioritization scenarios. (a) % infections averted in the whole population after five years and (b) PrEP efficiency (life-years gained per 100 person-years on PrEP) measured in the whole population over 20 years, for the same total number of person-years on PrEP for different FSW prioritization scenarios. Bars show median and error bars are 95% credible interval across 115 parameter sets.

The proportion of IA and PrEP efficiency were roughly halved when the same amount of PrEP was distributed amongst all FSWs or high-condom FSWs, compared with low-condom FSWs (Figure 5).

To completely negate the beneficial impact of PrEP, a 50% relative reduction in condom use by those individuals using PrEP was needed when prioritizing PrEP to all FSWs, compared with a 90% relative reduction in condom use when PrEP was prioritized to HR-MSM or low-condom FSWs (Supplementary Figure 10).

### Scenario with lower baseline condom use

**Figure 6:**
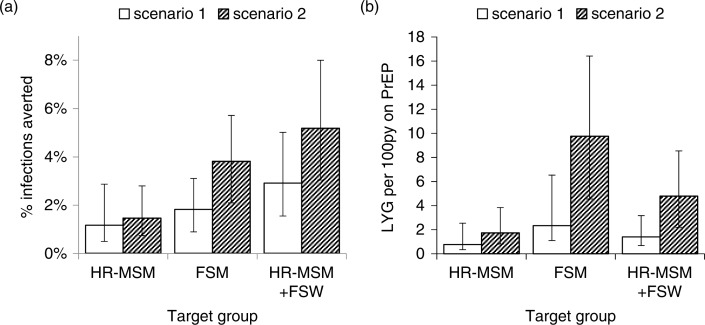
PrEP impact and efficiency for the lower condom use scenario. Impact and efficiency of prioritized PrEP interventions if there had been lower condom use amongst FSWs with their clients and MSM in Bangalore after 2003 (Scenario 2) compared to projections for the baseline scenario with observed levels of condom use (Scenario 1). More details for Scenario 2 are included in the main text and Supplementary File. (a) Impact in terms of % infections averted in the whole population after five years, and (b) efficiency in terms of life-years gained per 100 person-years on PrEP after 20 years, when a PrEP intervention reaches 60% of the prioritized group, with 50% adherence, for the prioritized groups shown.

When lower levels of condom use were assumed post-2003 (Scenario 2), the overall impact and efficiency of PrEP in the whole Bangalore population were predicted to be greater than in our original scenario, particularly when FSWs were prioritized (Figure 6). The percentage of IA in the whole population after five years was 26% greater in the lower condom use scenario compared to the baseline scenario when prioritizing HR-MSM, and 2.1-fold greater when prioritizing FSWs; efficiency after 20 years was 2.3-fold and 4.2-fold greater when prioritizing HR-MSM or FSWs, respectively.

## Discussion

We found that PrEP prioritized to FSWs or HR-MSM in Bangalore could substantially reduce HIV incidence amongst the prioritized group, preventing 22 to 25% of infections over the first five years of an intervention achieving 60% PrEP coverage of the prioritized group with 50% adherence. However, we predict a much smaller impact in the whole Bangalore population, due to higher levels of HIV transmission now occurring outside these high-risk groups. This is reflected in the lower PAF of commercial sex and sex between men predicted after 2016, and higher PAF for regular partnerships. Other modelling studies have also suggested that the PAF for commercial sex has declined over time in West/Central Africa [37,38] and southern India [6], as condom use in commercial sex increased and greater HIV transmission occurred outside key populations.

Sixty percent coverage of key populations requires high PrEP uptake rates, which may be feasible given high willingness to use PrEP among Indian MSM and FSWs [39].

A greater population-level impact was seen if FSWs were prioritized rather than HR-MSM, due to FSWs and their clients having more sexual interactions with the low-risk population than MSM, so more infections are averted outside key populations. We saw an almost additive effect of prioritizing both FSWs and HR-MSM together, due to the small overlap in their sexual networks as evidenced by the small degree to which FSWs contribute to MSM HIV transmission and vice versa.

Due to the greater number of infections prevented outside the prioritized population when FSWs were given PrEP rather than HR-MSM, the efficiency of PrEP was greater when prioritizing FSWs, although still rather low. The most efficient intervention – prioritizing FSWs with lower condom use – only achieved 4 LYG/100 person-years on PrEP over 20 years, which is unlikely to be cost-effective, given the high costs of PrEP interventions (cheapest estimate 95 US$/person/year [40], giving ≥US$2375/LYG). Another study modelling PrEP for FSWs in South India also found low efficiency, attributed to high levels of FSW condom use [41]. Another modelling study predicted low impact of PrEP for FSWs using condoms frequently in commercial sex [42]. HIV prevention cost-effectiveness studies have suggested that it is most cost-effective to first implement behaviour change interventions and early ART, with the subsequent addition of PrEP not always being cost-effective [43–45]. However, as local epidemics head towards elimination, it may become necessary to shift the focus from cost-effectiveness thresholds towards achieving elimination efficiently.

The most efficient PrEP strategy in this setting was to only give PrEP to FSWs with lower condom use. However, due to the small size of this group, this strategy would only give a small impact on the overall epidemic. Studies estimating the cost-effectiveness of PrEP for MSM in Australia [46] and Peru [47] have similarly found that, although it is more cost-effective, the prioritization of small, higher-risk groups for PrEP has limited population-level impact.

We predicted that the Bangalore HIV epidemic is close to elimination without PrEP, using a threshold of population incidence below 1/1000 person-years [48], and that, while PrEP for FSWs or MSM gives large incidence reductions among the prioritized population, it only slightly increases the likelihood and speed of reaching elimination. We assume that high levels of condom use are maintained, which will be crucial for achieving elimination. Future analyses should investigate whether elimination could be accelerated by additionally intervening among clients or regular partners [49].

We predicted that expanded ART eligibility would only slightly reduce the impact and efficiency of PrEP interventions, perhaps due to low HIV testing rates amongst the general population, where most new infections occur.

We predicted that large reductions in condom use (<50% for FSW, <90% for MSM) could occur amongst individuals taking PrEP (with 50% adherence) without offsetting the full benefits of PrEP, and so although condom use should continue to be promoted, condom migration is unlikely to negate the effects of PrEP in this setting. This agrees with MSM studies [46,47], which suggested >80% reduction in condom use by MSM on PrEP would be needed to cancel out PrEP benefits. However, a study among FSWs in India suggested that a 15% reduction in condom use by FSWs taking PrEP could negate the impact of PrEP [41].

Finally, we predicted greater impact and efficiency of PrEP in settings with lower condom use and higher HIV prevalence, suggesting that prioritized PrEP could be valuable in settings where condom use remains persistently low despite efforts to scale it up; this agrees with earlier modelling studies of topical PrEP [50].

### Strengths and limitations

Our model made use of extensive setting-specific data from key populations, which should improve the reliability of our results; however, the data may not be fully representative of local key populations, since the sampling methods used were designed to capture higher-risk MSM, and participation rates were relatively low, particularly among clients. If the individuals sampled were at higher risk than the wider key population groups, we may have overestimated HIV incidence and PrEP impact. Our fitting method ensured that considerable uncertainty in many of the model parameters was taken into account, and so our findings should be robust to this uncertainty. In the absence of data on PrEP retention, we assumed 20% annual dropout; lower retention would reduce the PrEP coverage that can be achieved, reducing impact. Our model did not stratify the FSW and MSM populations by sexual activity, and so we were not able to assess the possible impact of prioritizing PrEP to individuals with higher levels of sexual activity, which could improve PrEP efficiency. Cost-effectiveness could not be assessed as cost data was not included.

## Conclusions

PrEP could have a substantial impact upon HIV incidence amongst FSWs and MSM in Bangalore, but is likely to have a minor impact at the general population level. Prioritizing PrEP to FSWs with sub-optimal condom use will improve efficiency but reduce overall population-level impact in this setting. In other settings with lower levels of condom use amongst key populations, the provision of PrEP to these high-risk populations may result in a greater general-population impact.

## Supplementary Material

Potential impact of pre-exposure prophylaxis for female sex workers and men who have sex with men in Bangalore, India: a mathematical modelling study
