# Supplementary material for: Potential impact of pre-exposure prophylaxis for female sex workers and men who have sex with men in Bangalore, India: a mathematical modelling study
Source: J Int AIDS Soc. 2016 Sep 7;19(1):20942. doi: 10.7448/IAS.19.1.20942 (PMC5016494; doi:10.7448/IAS.19.1.20942)
Supplement: Potential impact of pre-exposure prophylaxis for female sex workers and men who have sex with men in Bangalore, India: a mathematical modelling study [file JIAS-19-20942-s001.pdf]

Supplementary Material

Additional model details

Population groups

FSW clients – also referred to as 'clients' – are non-MSM clients of FSWs. MSM may also have commercial sex with FSWs in this model. Former MSM, FSWs and clients no longer engage in commercial sex or same-sex relationships. Low-risk males and females are not – and never have - engaged in commercial sex or same-sex relationships.

MSM, clients and FSW are recruited from the low-risk population at rates that keep the distribution by risk group constant in the absence of HIV. There is no direct replacement of AIDS-related deaths.

HR-MSM report more MSM partners than *panthi*/bisexuals, and are the MSM most likely to be contacted by existing interventions. The model reflects uncertainty around the relative sizes of the MSM groups; HR-MSM account for 15-60% of all MSM in different model runs.

By 2011, low-condom FSWs account for ~10% of modelled FSWs, and have per-sex-act condom use of ~70%, whereas high-condom FSWs have >99% per-sex-act condom use.

Balancing sex acts

Because of uncertainty in the *panthi*/bisexual population size and sexual activity, insertive and receptive MSM sex acts are balanced by varying the *panthi*/bisexual population size at baseline and subsequently varying the frequency of *panthi*/bisexual sex acts.

Sex acts between FSWs and clients are balanced by varying the size of the client population at baseline, and subsequently varying the frequency of client sex acts.

Infectivity and transmission assumptions

Increased transmission is associated with the acute and pre-AIDS stages of HIV infection, and sexual activity is assumed to cease in the AIDS stage (if not on ART).

HIV is transmitted between MSM through anal sex, between FSWs and clients/MSM through commercial vaginal and anal sex, and between men and women in any of the groups through anal and vaginal sex in non-commercial partnerships.

The model was coded in C++ and the equations were solved numerically using an Euler algorithm with time-step 0.001 years.

Model equations

Equations for active MSM, FSWs and clients, and for the low-risk general population (i = 0-6, 12, 13)

$$\frac{d}{dt}(X_{1,\mathrm{i}}) = p_i\sigma_i - X_{1,i}(\lambda_{1,0} + \mu_i + \chi_i + \psi_i + \eta_i)$$

$$\frac{d}{dt}(X_{2,\mathrm{i}}) = (1 - p_i)\sigma_i + \nu X_{3,i} - X_{2,i}(\lambda_{1,0} + \mu_i + \tau_i r_i + \chi_i + \psi_i + \eta_i)$$

$$\frac{d}{dt}(X_{3,\mathrm{i}}) = \tau_i r_i X_{2,i} - X_{3,i}(\lambda_{1,1} + \mu_i + \nu + \chi_i + \psi_i + \eta_i)$$

$$\frac{d}{dt}(\mathrm{Y}_{1,1,\mathrm{i}}) = \lambda_{1,0} X_{1,i} - Y_{1,1,i}(\gamma_1 + \mu_i + \chi_i + \psi_i)$$

$$\frac{d}{dt}(\mathrm{Y}_{2,1,\mathrm{i}}) = \gamma_1 Y_{1,1,i} - Y_{2,1,i}(\gamma_2 + \mu_i + \chi_i + \psi_i)$$

$$\frac{d}{dt}(\mathrm{Y}_{3,1,\mathrm{i}}) = \gamma_2 Y_{2,1,i} - Y_{3,1,i}(\gamma_3 + \mu_i + \chi_i + \psi_i)$$

$$\frac{d}{dt}(\mathrm{Y}_{4,1,\mathrm{i}}) = \gamma_3 Y_{3,1,i} - Y_{4,1,i}(\gamma_4 + \mu_i + \chi_i + \psi_i)$$

$$\frac{d}{dt}(\mathrm{Y}_{5,1,\mathrm{i}}) = \gamma_4 Y_{4,1,i} - Y_{5,1,i}(\gamma_5 + a_{1,5} + \mu_i + \chi_i + \psi_i)$$

$$\frac{d}{dt}(\mathrm{Y}_{6,1,\mathrm{i}}) = \gamma_5 Y_{5,1,i} - Y_{6,1,i}(\gamma_6 + a_{1,6} + \mu_i + \chi_i + \psi_i)$$

$$\frac{d}{dt}(\mathrm{Y}_{7,1,\mathrm{i}}) = \gamma_6 Y_{6,1,i} - Y_{7,1,i}(\gamma_7 + a_{1,7} + \mu_i + \chi_i + \psi_i)$$

$$\frac{d}{dt}(\mathrm{Y}_{1,2,\mathrm{i}}) = \lambda_{1,0} X_{2,i} + \omega Y_{1,3,i} - Y_{1,2,i}(\gamma_1 + \varepsilon_i + \mu_i + \chi_i + \psi_i)$$

$$\frac{d}{dt}(\mathrm{Y}_{2,2,\mathrm{i}}) = \gamma_1 Y_{1,2,i} + \omega Y_{2,3,i} - Y_{2,2,i}(\gamma_2 + \varepsilon_i + \mu_i + \chi_i + \psi_i)$$

$$\frac{d}{dt}(\mathrm{Y}_{3,2,\mathrm{i}}) = \gamma_2 Y_{2,2,i} + \omega Y_{3,3,i} - Y_{3,2,i}(\gamma_3 + \varepsilon_i + \mu_i + \chi_i + \psi_i)$$

$$\frac{d}{dt}(\mathrm{Y}_{4,2,\mathrm{i}}) = \gamma_3 Y_{3,2,i} + \omega Y_{4,3,i} - Y_{4,2,i}(\gamma_4 + \varepsilon_i + \mu_i + \chi_i + \psi_i)$$

$$\frac{d}{dt}(\mathrm{Y}_{5,2,\mathrm{i}}) = \gamma_4 Y_{4,2,i} + \omega Y_{5,3,i} - Y_{5,2,i}(\gamma_5 + a_{1,5} + \varepsilon_i + \mu_i + \chi_i + \psi_i)$$

$$\frac{d}{dt}(\mathrm{Y}_{6,2,\mathrm{i}}) = \gamma_5 Y_{5,2,i} + \omega Y_{6,3,i} - Y_{6,2,i}(\gamma_6 + a_{1,6} + \varepsilon_i + \mu_i + \chi_i + \psi_i)$$

$$\frac{d}{dt}(\mathrm{Y}_{7,2,\mathrm{i}}) = \gamma_6 Y_{6,2,i} + \omega Y_{7,3,i} - Y_{7,2,i}(\gamma_7 + a_{1,7} + \varepsilon_i + \mu_i + \chi_i + \psi_i)$$

$$\frac{d}{dt}(\mathrm{Y}_{1,3,\mathrm{i}}) = \lambda_{1,1}X_{3,i} + \varepsilon_i Y_{1,2,i} - Y_{1,3,i}(\gamma_1 + a_{2,1,i} + \omega + \mu_i + \chi_i + \psi_i)$$

$$\frac{d}{dt}(\mathrm{Y}_{2,3,\mathrm{i}}) = \gamma_1 Y_{1,3,i} + \varepsilon_i Y_{2,2,i} - Y_{2,3,i}(\gamma_2 + a_{2,2,i} + \omega + \mu_i + \chi_i + \psi_i)$$

$$\frac{d}{dt}(\mathrm{Y}_{3,3,\mathrm{i}}) = \gamma_2 Y_{2,3,i} + \varepsilon_i Y_{3,2,i} - Y_{3,3,i}(\gamma_3 + a_{2,3,i} + \omega + \mu_i + \chi_i + \psi_i)$$

$$\frac{d}{dt}(\mathrm{Y}_{4,3,\mathrm{i}}) = \gamma_3 Y_{3,3,i} + \varepsilon_i Y_{4,2,i} - Y_{4,3,i}(\gamma_4 + a_{2,4,i} + \omega + \mu_i + \chi_i + \psi_i)$$

$$\frac{d}{dt}(\mathrm{Y}_{5,3,\mathrm{i}}) = \gamma_4 Y_{4,3,i} + \varepsilon_i Y_{5,2,i} - Y_{5,3,i}(\gamma_5 + a_{2,5,i} + \omega + \mu_i + \chi_i + \psi_i)$$

$$\frac{d}{dt}(\mathrm{Y}_{6,3,\mathrm{i}}) = \gamma_5 Y_{5,3,i} + \varepsilon_i Y_{6,2,i} - Y_{6,3,i}(\gamma_6 + a_{2,6,i} + \omega + \mu_i + \chi_i + \psi_i)$$

$$\frac{d}{dt}(\mathrm{Y}_{7,3,\mathrm{i}}) = \gamma_6 Y_{6,3,i} + \varepsilon_i Y_{7,2,i} - Y_{7,3,i}(\gamma_7 + a_{2,7,i} + \omega + \mu_i + \chi_i + \psi_i)$$

$$\frac{d}{dt}(\mathrm{Y}_{1,4,\mathrm{i}}) = a_{2,1,i} Y_{1,3,i} - Y_{1,4,i}(\delta\gamma_1 + \phi + \mu_i + \chi_i + \psi_i)$$

$$\frac{d}{dt}(\mathrm{Y}_{2,4,\mathrm{i}}) = \delta\gamma_1 Y_{1,4i} + a_{2,2,i} Y_{2,3,i} - Y_{2,4,i}(\delta\gamma_2 + \phi + \mu_i + \chi_i + \psi_i)$$

$$\frac{d}{dt}(\mathrm{Y}_{3,4,\mathrm{i}}) = \delta\gamma_2 Y_{2,4,i} + a_{2,3,i} Y_{3,3,i} - Y_{3,4,i}(\delta\gamma_3 + \phi + \mu_i + \chi_i + \psi_i)$$

$$\frac{d}{dt}(\mathrm{Y}_{4,4,\mathrm{i}}) = \delta\gamma_3 Y_{3,4,i} + a_{2,4,i} Y_{4,3,i} - Y_{4,4,i}(\delta\gamma_4 + \phi + \mu_i + \chi_i + \psi_i)$$

$$\frac{d}{dt}(\mathrm{Y}_{5,4,\mathrm{i}}) = \delta\gamma_4 Y_{4,4,i} + a_{2,5,i} Y_{5,3,i} + a_{1,5}(Y_{5,1,i} + Y_{5,2,i} + Y_{5,5,i}) - Y_{5,4,i}(\delta\gamma_5 + \phi + \mu_i + \chi_i + \psi_i)$$

$$\frac{d}{dt}(\mathrm{Y}_{6,4,\mathrm{i}}) = \delta\gamma_5 Y_{5,4,i} + a_{2,6,i} Y_{6,3,i} + a_{1,6}(Y_{6,1,i} + Y_{6,2,i} + Y_{6,5,i}) - Y_{6,4,i}(\delta\gamma_6 + \phi + \mu_i + \chi_i + \psi_i)$$

$$\frac{d}{dt}(\mathrm{Y}_{7,4,\mathrm{i}}) = \delta\gamma_6 Y_{6,4,i} + a_{2,7,i} Y_{7,3,i} + a_{1,7}(Y_{7,1,i} + Y_{7,2,i} + Y_{7,5,i}) - Y_{7,4,i}(\delta\gamma_7 + \phi + \mu_i + \chi_i + \psi_i)$$

$$\frac{d}{dt}(\mathrm{Y}_{1,5,\mathrm{i}}) = \phi Y_{1,4,i} - Y_{1,5,i}(\gamma_1 + \mu_i + \chi_i + \psi_i)$$

$$\frac{d}{dt}(\mathrm{Y}_{2,5,\mathrm{i}}) = \gamma_1 Y_{1,5,i} + \phi Y_{2,4,i} - Y_{2,5,i}(\gamma_2 + \mu_i + \chi_i + \psi_i)$$

$$\frac{d}{dt}(\mathrm{Y}_{3,5,\mathrm{i}}) = \gamma_2 Y_{2,5,i} + \phi Y_{3,4,i} - Y_{3,5,i}(\gamma_3 + \mu_i + \chi_i + \psi_i)$$

$$\frac{d}{dt}(\mathrm{Y}_{4,5,\mathrm{i}}) = \gamma_3 Y_{3,5,i} + \phi Y_{4,4,i} - Y_{4,5,i}(\gamma_4 + \mu_i + \chi_i + \psi_i)$$

$$\frac{d}{dt}(\mathrm{Y}_{5,5,\mathrm{i}}) = \gamma_4 Y_{4,5,i} + \phi Y_{5,4,i} - Y_{5,5,i}(\gamma_5 + a_{1,5} + \mu_i + \chi_i + \psi_i)$$

$$\frac{d}{dt}(\mathrm{Y}_{6,5,\mathrm{i}}) = \gamma_5 Y_{5,5,i} + \phi Y_{6,4,i} - Y_{6,5,i}(\gamma_6 + a_{1,6} + \mu_i + \chi_i + \psi_i)$$

$$\frac{d}{dt}(\mathrm{Y}_{7,5,\mathrm{i}}) = \gamma_6 Y_{6,5,i} + \phi Y_{7,4,i} - Y_{7,5,i}(\gamma_7 + a_{1,7} + \mu_i + \chi_i + \psi_i)$$

Equations for former MSM, FSWs and clients (i = 7-11):

$$\frac{d}{dt}(X_{1,\mathrm{i}}) = \psi_{i-6}X_{1,i-6} - X_{1,i}(\lambda_{1,0} + \mu_i)$$

$$\frac{d}{dt}(X_{2,\mathrm{i}}) = \psi_{i-6}X_{2,i-6} + \psi_{i-6}X_{3,i-6} - X_{2,i}(\lambda_{1,0} + \mu_i)$$

$$\frac{d}{dt}(X_{3,\mathrm{i}}) = 0$$

$$\frac{d}{dt}(\mathrm{Y}_{1,1,\mathrm{i}}) = \psi_{i-6}Y_{1,1,i-6} + \lambda_{1,0}X_{1,i} - Y_{1,1,i}(\gamma_1 + \mu_i)$$

$$\frac{d}{dt}(\mathrm{Y}_{2,1,\mathrm{i}}) = \psi_{i-6}Y_{2,1,i-6} + \gamma_1 Y_{1,1,i} - Y_{2,1,i}(\gamma_2 + \mu_i)$$

$$\frac{d}{dt}(\mathrm{Y}_{3,1,\mathrm{i}}) = \psi_{i-6}Y_{3,1,i-6} + \gamma_2 Y_{2,1,i} - Y_{3,1,i}(\gamma_3 + \mu_i)$$

$$\frac{d}{dt}(\mathrm{Y}_{4,1,\mathrm{i}}) = \psi_{i-6}Y_{4,1,i-6} + \gamma_3 Y_{3,1,i} - Y_{4,1,i}(\gamma_4 + \mu_i)$$

$$\frac{d}{dt}(\mathrm{Y}_{5,1,\mathrm{i}}) = \psi_{i-6}Y_{5,1,i-6} + \gamma_4 Y_{4,1,i} - Y_{5,1,i}(\gamma_5 + a_{1,5} + \mu_i)$$

$$\frac{d}{dt}(\mathrm{Y}_{6,1,\mathrm{i}}) = \psi_{i-6}Y_{6,1,i-6} + \gamma_5 Y_{5,1,i} - Y_{6,1,i}(\gamma_6 + a_{1,6} + \mu_i)$$

$$\frac{d}{dt}(\mathrm{Y}_{7,1,\mathrm{i}}) = \psi_{i-6}Y_{7,1,i-6} + \gamma_6 Y_{6,1,i} - Y_{7,1,i}(\gamma_7 + a_{1,7} + \mu_i)$$

$$\frac{d}{dt}(\mathrm{Y}_{1,2,\mathrm{i}}) = \psi_{i-6}Y_{1,2,i-6} + \lambda_{1,0}X_{2,i} + \omega Y_{1,3,i} - Y_{1,2,i}(\gamma_1 + \varepsilon_i + \mu_i)$$

$$\frac{d}{dt}(\mathrm{Y}_{2,2,\mathrm{i}}) = \psi_{i-6}Y_{2,2,i-6} + \gamma_1 Y_{1,2,i} + \omega Y_{2,3,i} - Y_{2,2,i}(\gamma_2 + \varepsilon_i + \mu_i)$$

$$\frac{d}{dt}(\mathrm{Y}_{3,2,\mathrm{i}}) = \psi_{i-6}Y_{3,2,i-6} + \gamma_2 Y_{2,2,i} + \omega Y_{3,3,i} - Y_{3,2,i}(\gamma_3 + \varepsilon_i + \mu_i)$$

$$\frac{d}{dt}(\mathrm{Y}_{4,2,\mathrm{i}}) = \psi_{i-6}Y_{4,2,i-6} + \gamma_3 Y_{3,2,i} + \omega Y_{4,3,i} - Y_{4,2,i}(\gamma_4 + \varepsilon_i + \mu_i)$$

$$\frac{d}{dt}(\mathrm{Y}_{5,2,\mathrm{i}}) = \psi_{i-6}Y_{5,2,i-6} + \gamma_4 Y_{4,2,i} + \omega Y_{5,3,i} - Y_{5,2,i}(\gamma_5 + a_{1,5} + \varepsilon_i + \mu_i)$$

$$\frac{d}{dt}(\mathrm{Y}_{6,2,\mathrm{i}}) = \psi_{i-6}Y_{6,2,i-6} + \gamma_5 Y_{5,2,i} + \omega Y_{6,3,i} - Y_{6,2,i}(\gamma_6 + a_{1,6} + \varepsilon_i + \mu_i)$$

$$\frac{d}{dt}(\mathrm{Y}_{7,2,\mathrm{i}}) = \psi_{i-6}Y_{7,2,i-6} + \gamma_6 Y_{6,2,i} + \omega Y_{7,3,i} - Y_{7,2,i}(\gamma_7 + a_{1,7} + \varepsilon_i + \mu_i)$$

$$\frac{d}{dt}(\mathrm{Y}_{1,3,\mathrm{i}}) = \psi_{i-6}Y_{1,3,i-6} + \varepsilon_i Y_{1,2,i} - Y_{1,3,i}(\gamma_1 + a_{2,1,i} + \omega + \mu_i)$$

$$\frac{d}{dt}(\mathrm{Y}_{2,3,\mathrm{i}}) = \psi_{i-6}Y_{2,3,i-6} + \gamma_1 Y_{1,3,i} + \varepsilon_i Y_{2,2,i} - Y_{2,3,i}(\gamma_2 + a_{2,2,i} + \omega + \mu_i)$$

$$\frac{d}{dt}(\mathrm{Y}_{3,3,\mathrm{i}}) = \psi_{i-6}Y_{3,3,i-6} + \gamma_2 Y_{2,3,i} + \varepsilon_i Y_{3,2,i} - Y_{3,3,i}(\gamma_3 + a_{2,3,i} + \omega + \mu_i)$$

$$\frac{d}{dt}(\mathrm{Y}_{4,3,\mathrm{i}}) = \psi_{i-6}Y_{4,3,i-6} + \gamma_3 Y_{3,3,i} + \varepsilon_i Y_{4,2,i} - Y_{4,3,i}(\gamma_4 + a_{2,4,i} + \omega + \mu_i)$$

$$\frac{d}{dt}(\mathrm{Y}_{5,3,\mathrm{i}}) = \psi_{i-6}Y_{5,3,i-6} + \gamma_4 Y_{4,3,i} + \varepsilon_i Y_{5,2,i} - Y_{5,3,i}(\gamma_5 + a_{2,5,i} + \omega + \mu_i)$$

$$\frac{d}{dt}(\mathrm{Y}_{6,3,\mathrm{i}}) = \psi_{i-6}Y_{6,3,i-6} + \gamma_5 Y_{5,3,i} + \varepsilon_i Y_{6,2,i} - Y_{6,3,i}(\gamma_6 + a_{2,6,i} + \omega + \mu_i)$$

$$\frac{d}{dt}(\mathrm{Y}_{7,3,\mathrm{i}}) = \psi_{i-6}Y_{7,3,i-6} + \gamma_6 Y_{6,3,i} + \varepsilon_i Y_{7,2,i} - Y_{7,3,i}(\gamma_7 + a_{2,7,i} + \omega + \mu_i)$$

$$\frac{d}{dt}(\mathrm{Y}_{1,4,\mathrm{i}}) = \psi_{i-6}Y_{1,4,i-6} + a_{2,1,i}Y_{1,3,i} - Y_{1,4,i}(\delta\gamma_1 + \phi + \mu_i)$$

$$\frac{d}{dt}(\mathrm{Y}_{2,4,\mathrm{i}}) = \psi_{i-6}Y_{2,4,i-6} + \delta\gamma_1 Y_{1,4i} + a_{2,2,i}Y_{2,3,i} - Y_{2,4,i}(\delta\gamma_2 + \phi + \mu_i)$$

$$\frac{d}{dt}(\mathrm{Y}_{3,4,\mathrm{i}}) = \psi_{i-6}Y_{3,4,i-6} + \delta\gamma_2 Y_{2,4,i} + a_{2,3,i}Y_{3,3,i} - Y_{3,4,i}(\delta\gamma_3 + \phi + \mu_i)$$

$$\frac{d}{dt}(\mathrm{Y}_{4,4,\mathrm{i}}) = \psi_{i-6}Y_{4,4,i-6} + \delta\gamma_3 Y_{3,4,i} + a_{2,4,i}Y_{4,3,i} - Y_{4,4,i}(\delta\gamma_4 + \phi + \mu_i)$$

$$\frac{d}{dt}(\mathrm{Y}_{5,4,\mathrm{i}}) = \psi_{i-6}Y_{5,4,i-6} + \delta\gamma_4 Y_{4,4,i} + a_{2,5,i}Y_{5,3,i} + a_{1,5}(Y_{5,1,i} + Y_{5,2,i} + Y_{5,5,i}) - Y_{5,4,i}(\delta\gamma_5 + \phi + \mu_i)$$

$$\frac{d}{dt}(\mathrm{Y}_{6,4,\mathrm{i}}) = \psi_{i-6}Y_{6,4,i-6} + \delta\gamma_5 Y_{5,4,i} + a_{2,6,i}Y_{6,3,i} + a_{1,6}(Y_{6,1,i} + Y_{6,2,i} + Y_{6,5,i}) - Y_{6,4,i}(\delta\gamma_6 + \phi + \mu_i)$$

$$\frac{d}{dt}(\mathrm{Y}_{7,4,\mathrm{i}}) = \psi_{i-6}Y_{7,4,i-6} + \delta\gamma_6 Y_{6,4,i} + a_{2,7,i}Y_{7,3,i} + a_{1,7}(Y_{7,1,i} + Y_{7,2,i} + Y_{7,5,i}) - Y_{7,4,i}(\delta\gamma_7 + \phi + \mu_i)$$

$$\frac{d}{dt}(\mathrm{Y}_{1,5,\mathrm{i}}) = \psi_{i-6}Y_{1,5,i-6} + \phi Y_{1,4,i} - Y_{1,5,i}(\gamma_1 + \mu_i)$$

$$\frac{d}{dt}(\mathrm{Y}_{2,5,\mathrm{i}}) = \psi_{i-6}Y_{2,5,i-6} + \gamma_1 Y_{1,5,i} + \phi Y_{2,4,i} - Y_{2,5,i}(\gamma_2 + \mu_i)$$

$$\frac{d}{dt}(\mathrm{Y}_{3,5,\mathrm{i}}) = \psi_{i-6}Y_{3,5,i-6} + \gamma_2 Y_{2,5,i} + \phi Y_{3,4,i} - Y_{3,5,i}(\gamma_3 + \mu_i)$$

$$\frac{d}{dt}(\mathrm{Y}_{4,5,\mathrm{i}}) = \psi_{i-6}Y_{4,5,i-6} + \gamma_3 Y_{3,5,i} + \phi Y_{4,4,i} - Y_{4,5,i}(\gamma_4 + \mu_i)$$

$$\frac{d}{dt}(\mathrm{Y}_{5,5,\mathrm{i}}) = \psi_{i-6}Y_{5,5,i-6} + \gamma_4 Y_{4,5,i} + \phi Y_{5,4,i} - Y_{5,5,i}(\gamma_5 + a_{1,5} + \mu_i)$$

$$\frac{d}{dt}(\mathrm{Y}_{6,5,\mathrm{i}}) = \psi_{i-6}Y_{6,5,i-6} + \gamma_5 Y_{5,5,i} + \phi Y_{6,4,i} - Y_{6,5,i}(\gamma_6 + a_{1,6} + \mu_i)$$

$$\frac{d}{dt}(\mathrm{Y}_{7,5,\mathrm{i}}) = \psi_{i-6}Y_{7,5,i-6} + \gamma_6 Y_{6,5,i} + \phi Y_{7,4,i} - Y_{7,5,i}(\gamma_7 + a_{1,7} + \mu_i)$$

Sexual mixing in the model

Insertive and receptive anal sex acts between MSM groups are distributed separately in proportion to the total number of receptive and insertive anal sex acts offered by each MSM identity group. The probability that MSM in group i have a receptive act with MSM in group j (given that they have a receptive sex act) is given by:

$$\rho_{Rec,i,j} = \frac{N_j c_j x_j}{N_i c_i x_i + N_j c_j x_j + N_k c_k x_k}$$

Where N_j is the total number of MSM in group j, c_j is the total number of anal sex acts per year per person for MSM in group j and x_j is the proportion of sex acts which are insertive for MSM in group j.

Commercial sex acts between clients (MSM and non-MSM clients) and FSWs are distributed in proportion to the total number of commercial sex acts offered by each FSW risk group. The probability that a man in group i has a commercial sex act with an FSW in group l is given by:

$$\rho_{i,l} = \frac{N_l c_l}{N_l c_l + N_m c_m}$$

Sex acts in regular partnerships are distributed preferentially between groups of similar risk levels (low-risk population (including former high risk group members) versus current high risk groups), so that as many as possible of these sex acts occur between men and women in the low-risk population/ between current FSW/MSM/clients, with remaining sex acts being with partners of different risk levels. Within the same level of risk, regular partnership sex acts are distributed proportionately, as for commercial sex acts.

Force of infection

The force of infection, $\lambda_{i,j}$, for HIV negative individuals is determined by the prevalence and stage of HIV infection amongst their sexual partners, ART use by sexual partners, the number of partners they have sex with per year (c), the type of sex acts they have (vaginal or anal, insertive or receptive), the proportion of sex acts in which a condom is used (e_c), adherence to PrEP (e_f), the proportion of partnerships that are with individuals in risk group j ($\rho_{i,j}$), the probability of transmission per sex act with an HIV positive person (β), and the per-act reduction in transmission risk associated with correct condom use (f_c), PrEP use (f_p), and ART use (m_a).

For MSM having anal sex with other MSM:

$$\lambda_{i,0} = \sum_{j=0}^{j=2} \left(1 - e_c f_c(t)\right) \left(\left(\frac{c_i x_i \rho_{Ins,i,j} \kappa_a \beta_{rec,a} \left(m_1 \left(\sum_{s=1}^{s=3} Y_{1,s,j} + Y_{1,5,j} \right) + \sum_{t=2}^{t=5} \left(\sum_{s=1}^{s=3} Y_{t,s,j} + Y_{t,5,j} \right) + m_2 \left(\sum_{s=1}^{s=3} Y_{6,s,j} + Y_{6,5,j} \right) + m_a \left(\sum_{t=1}^{t=7} Y_{t,4,j} \right) \cdots}{\sum_{t=1}^{t=7} \sum_{s=1}^{s=5} Y_{t,s,j} + \sum_{s=1}^{s=3} X_{s,j}} \cdots \right. \right.$$

[illegible]

$$+ \left(\frac{c_i (1 - x_i) \rho_{Rec,i,j} \beta_{rec,a} \left(m_1 \left(\sum_{s=1}^{s=3} Y_{1,s} + Y_{1,5} \right) + \sum_{t=2}^{t=5} \left(\sum_{s=1}^{s=3} Y_{t,s} + Y_{t,5} \right) + m_2 \left(\sum_{s=1}^{s=3} Y_{6,s} + Y_{6,5} \right) + m_a \left(\sum_{t=1}^{t=7} Y_{t,4} \right) \right)}{\sum_{t=1}^{t=7} \sum_{s=1}^{s=5} Y_{t,s,j} + \sum_{s=1}^{s=3} X_{s,j}} \right) \Bigg) \Bigg)$$

$$\lambda_{i,1} = \lambda_{i,0} \left(1 - e_p f_p\right)$$

Model fitting

Due to the large parameter space explored, and the small impact of recent increases in ART coverage upon historical HIV prevalence trends, the model was fitted in two stages, first to HIV prevalence and then to ART coverage. In the first stage, parameters expected to predominantly affect only ART coverage (testing rates, linkage rates and dropout rates from ART and pre-ART care) were fixed at median values, while all other parameters were sampled from their ranges 1 million times using Latin Hypercube Sampling [1]. Only parameter combinations giving HIV prevalence projections within the 95% confidence intervals of HIV prevalence estimates for each key population were retained. In the second stage, each parameter set selected in stage 1 was used to fit ART coverage by varying testing, linkage and ART/pre-ART care dropout rates, while holding other parameters constant. For each parameter set selected in stage 1, we selected the combination of ART-coverage-related parameters giving the best least-squares fit to the number of people on ART (also fitting HIV prevalence and ART coverage). These parameter combinations were used for all subsequent analyses.

Supplementary figures

Figure S1: Model schematic

(a) HIV compartments

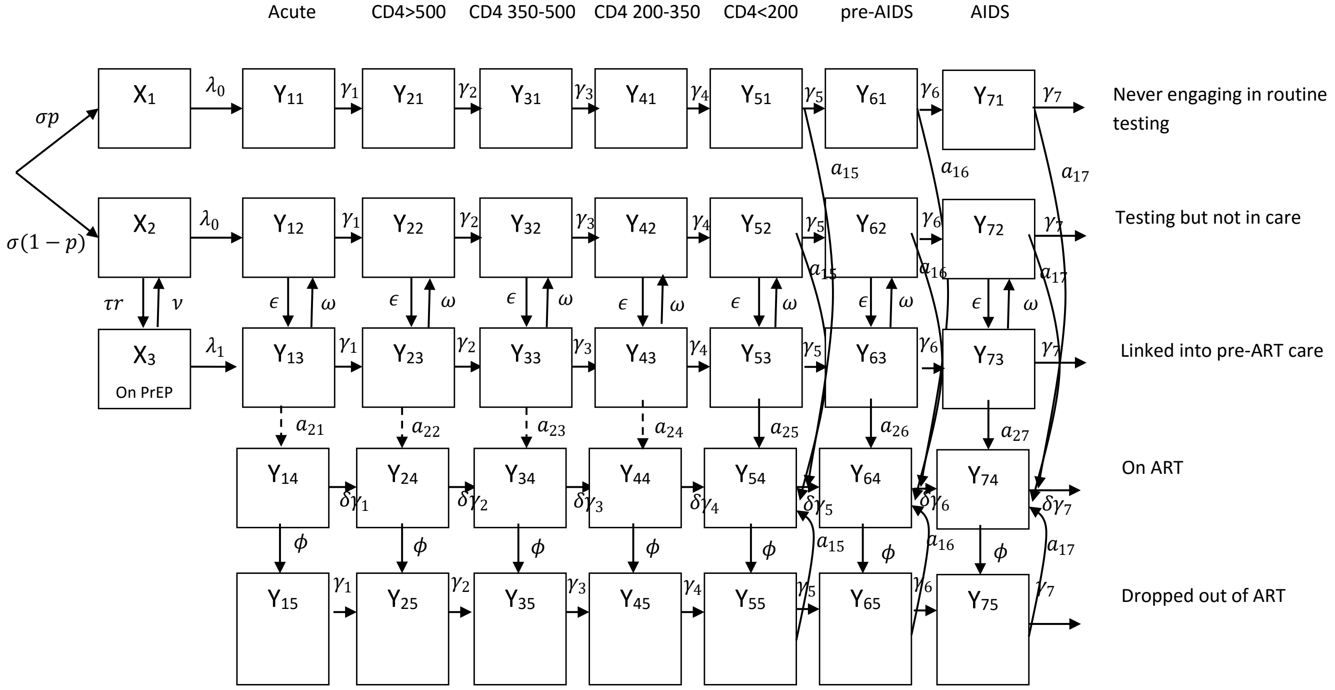

(b) Population groups and transitions

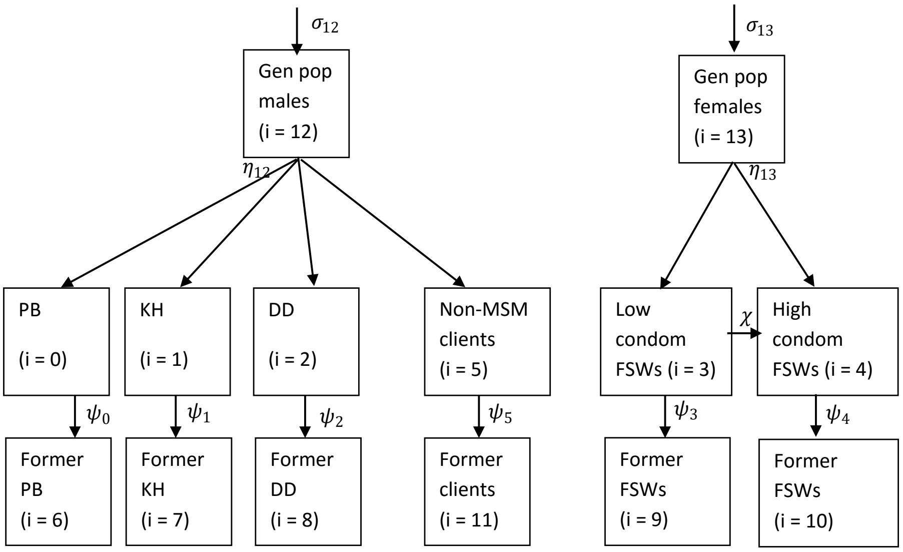

Figure S2. Condom use trends in scenario 2 in comparison with scenario 1. Condom use is the average % of sex acts in which condoms are used (a) by FSWs in commercial sex acts (over the whole FSW population) and (b) by MSM in sex acts with other men. Condom trends are shown for the best fit parameter set.

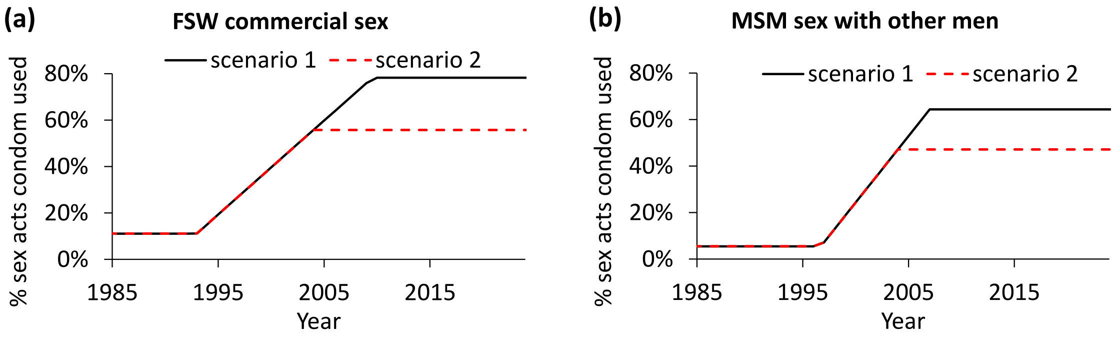

Figure S3. HIV prevalence trends in scenario 2 in comparison with scenario 1, for selected populations (a) all FSWs; (b) Kothi/hijra MSM; (c) the whole population.

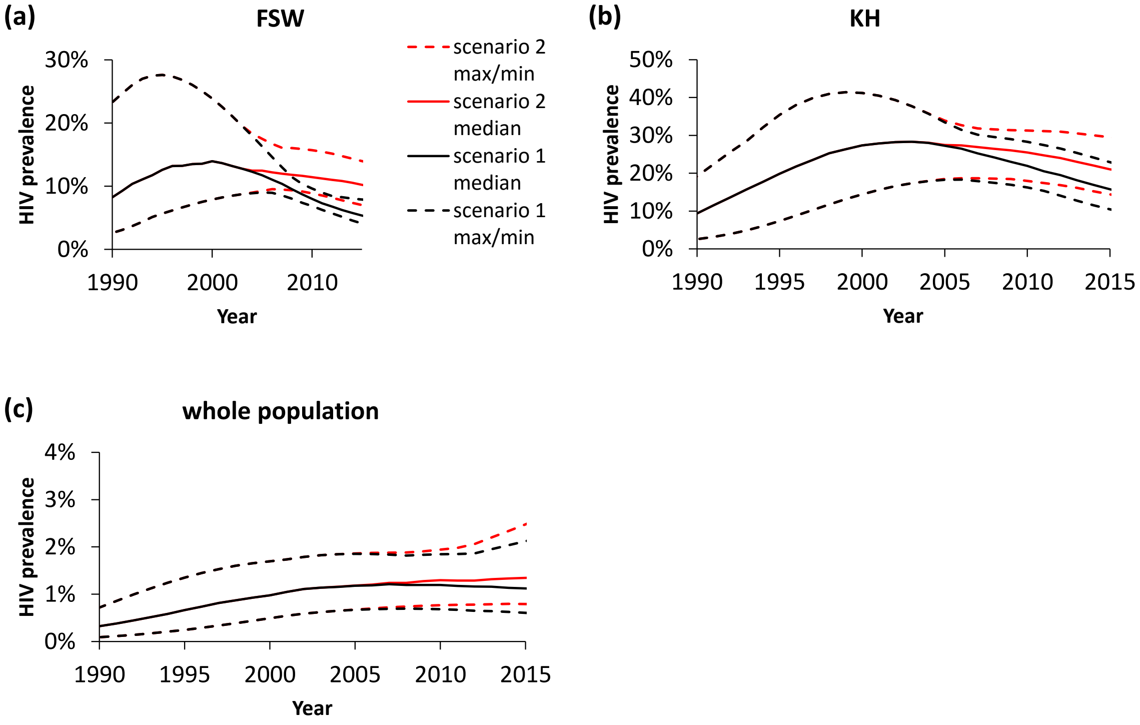

Figure S4. HIV prevalence time-trends for model fits, together with prevalence data used for fitting and validation, for (a) FSW, (b) non-MSM clients , (c) panthi/bisexual MSM, (d) kothi/hijra MSM, (e) double decker MSM, (f) the whole population, (g) men and (h) women. Lines show median (solid line), and 95% credible intervals (CrI; dashed lines) from 115 parameter sets. Credible intervals give the 2.5th and 97.5th percentiles of estimates across all parameter combinations. Filled diamonds: integrated behavioural and biological assessment (IBBA) survey data used for fitting. Open circles and crosses: ANC data not used for fitting [2]. Open diamonds: general population survey from Mysore, not used for fitting [3]. Error bars on data points are 95% confidence intervals. Note the different scales on the y-axes.

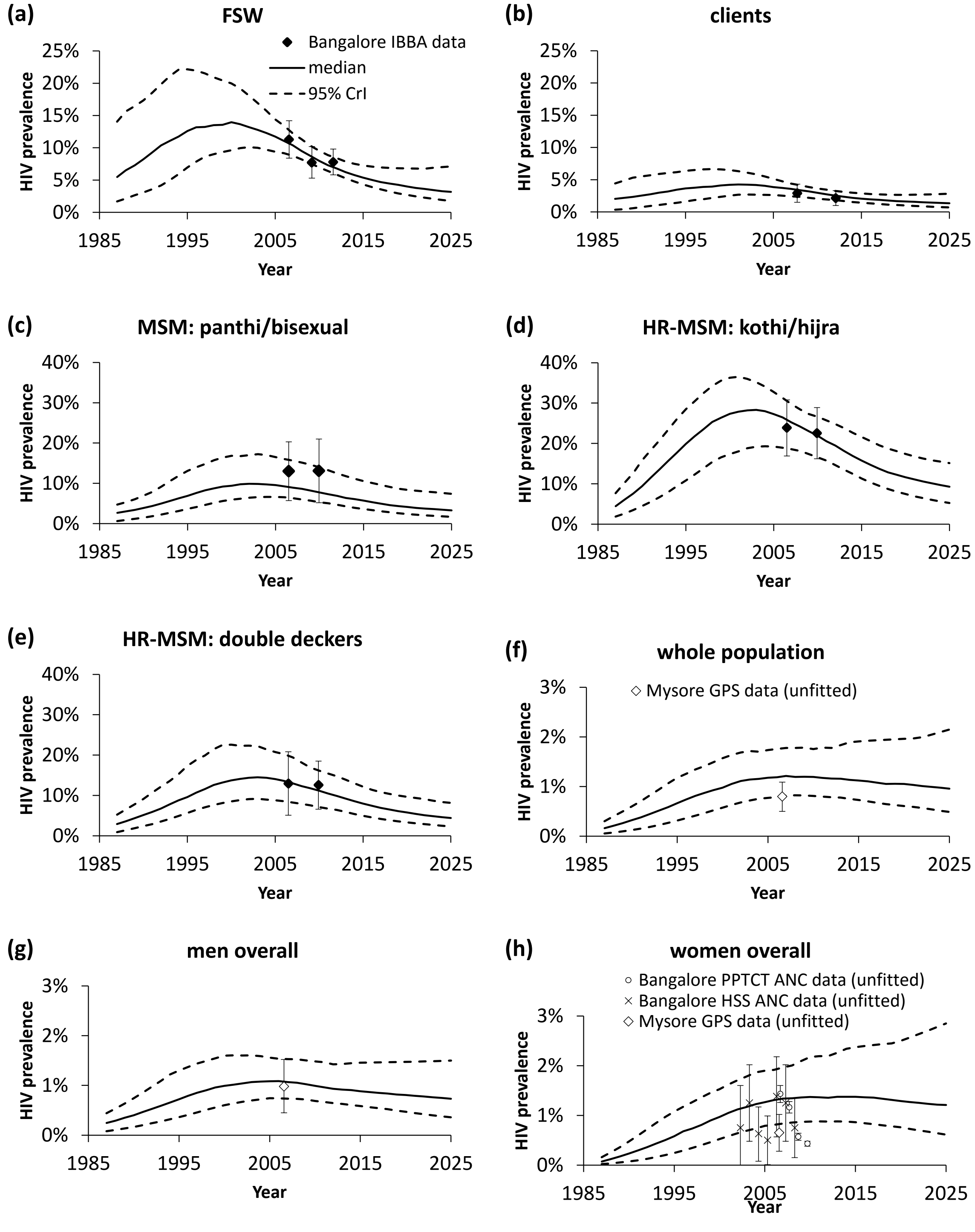

Figure S5. Population attributable fractions (a,b, d, e) in the whole population and (c, f) amongst MSM and FSW, for commercial sex and/or sex between MSM, or regular (non-commercial, heterosexual) partnerships for (a,b,c) scenario 1, projections with current observed levels of condom use and (d,e,f) scenario 2, where condom use is assumed to stay constant after 2003. Plots show the percentage of new infections averted amongst the group of interest over the time period shown, when transmission through commercial sex, sex between MSM or regular partnerships is stopped over that same time period. Points are median values and error bars are 95% credible intervals from 115 parameter sets.

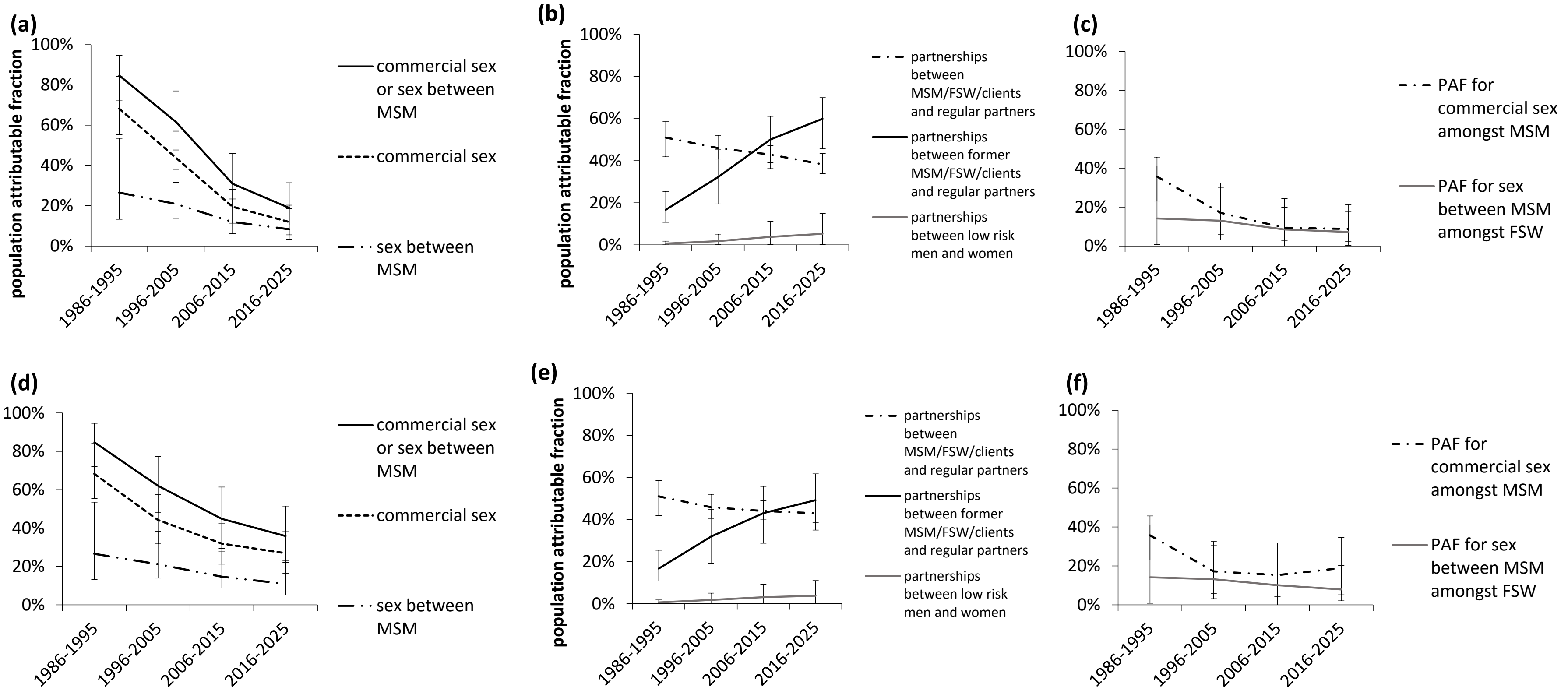

Figure S6. PrEP coverage over time amongst HIV-negative (a) FSW, (b) HR-MSM, when that group is prioritised. Lines are median from 115 parameters sets, calibrated to give 20%, 40% or 60% coverage of HIV-negative members of the prioritised group after 5 years. Time is years after the start of the PrEP intervention (beginning in 2017).

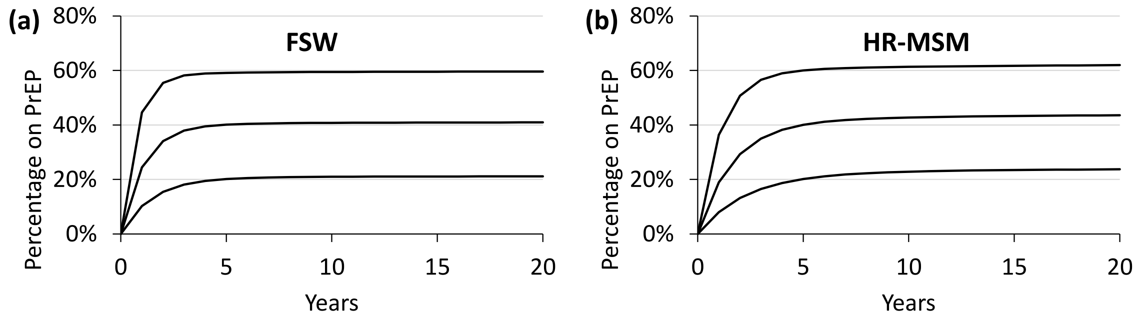

Figure S7. Incidence trends amongst (a) all FSW, (b) all MSM and (c) the whole population, without PrEP and with a PrEP intervention reaching 60% of all FSWs and HR-MSM, with adherence 30%, 50% or 75%. Results are shown for the best fit parameter set. The dotted horizontal line in (c) shows the elimination threshold (1 per 1000 people per year).

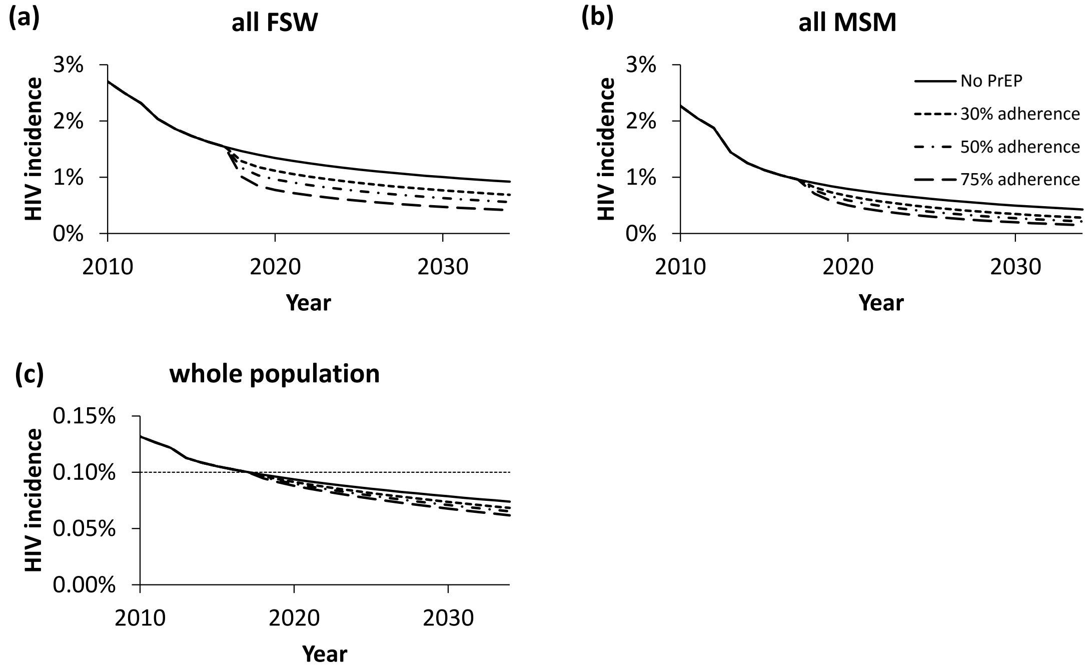

Figure S8. Absolute number of infections averted in the whole population over (a) 5 years and (b) 10 years, shown by risk group in which infections were averted (see key), when an intervention with 50% adherence and 60% coverage of the prioritised group is prioritised to the groups shown. Results are shown for the best fit parameter set. Note the different scales on the y-axis.

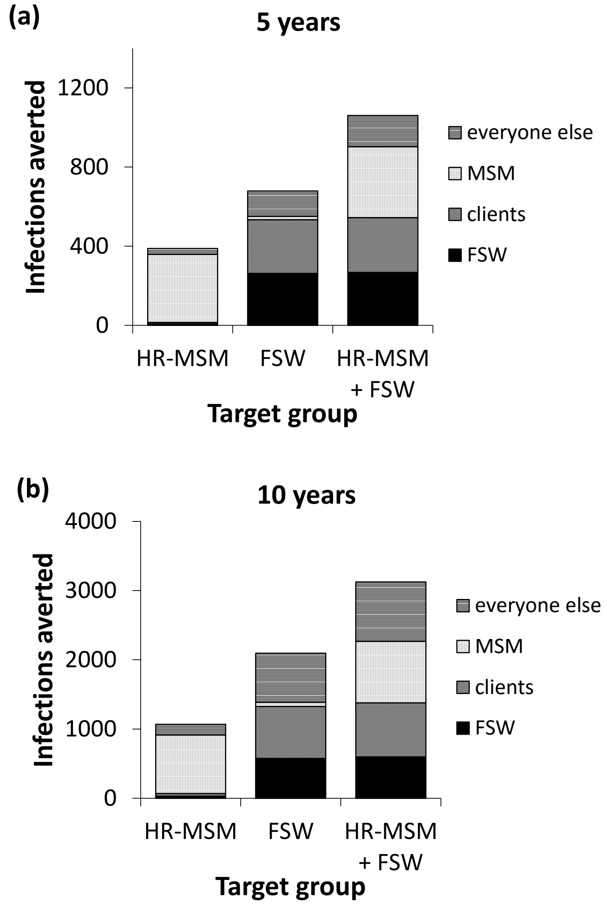

Figure S9. Impact and efficiency of prioritised PrEP interventions if ART guidelines were to change to expand ART access (all eligible for ART from 2017 onwards) compared to projections for the baseline scenario with continuation of current ART guidelines (eligibility with CD4 <350 cells/µl) (a) Impact in terms of % infections averted amongst FSWs over 5 years for PrEP interventions targeted to FSWS with PrEP adherence and coverage shown, (b) Impact in terms of % infections averted in the whole population after 5 years, (c) Efficiency in terms of life-years gained per 100 person-years on PrEP after 20 years, when a PrEP intervention reaches 60% of the prioritised group, with 50% adherence, for the prioritised groups shown.

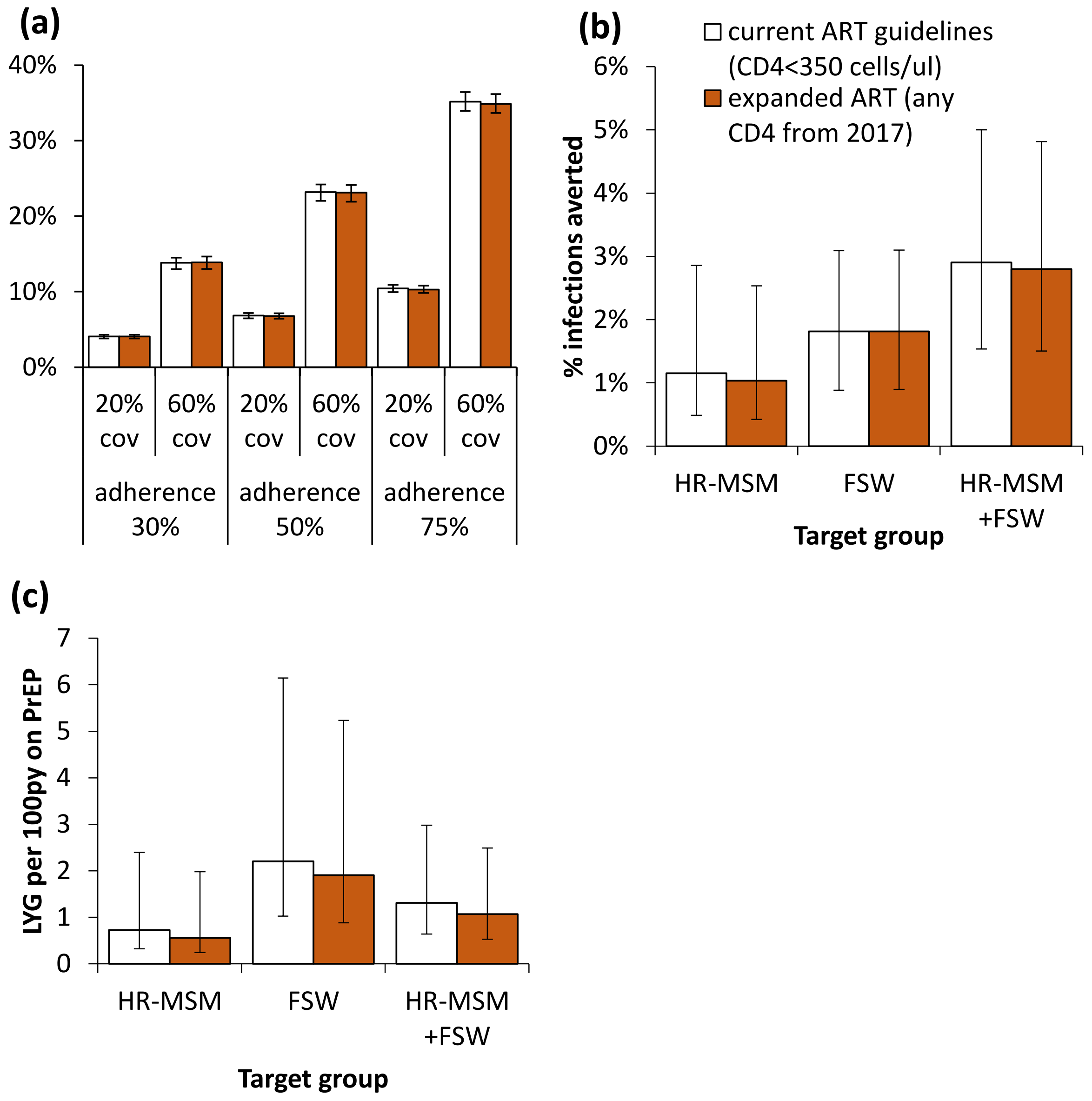

Figure S10. The effects of condom migration by PrEP users upon the impact of an intervention prioritised to (a) all FSWs, (b) HR-MSM or (c) low-condom FSWs. The impact, in terms of infections averted in the whole population after 5 years, is shown with existing levels of condom use (0% reduction), and with increasing levels of reduction in condom use, up to 100% reduction where condoms are not used at all, for a PrEP intervention reaching 60% of the prioritised group, with 50% adherence.

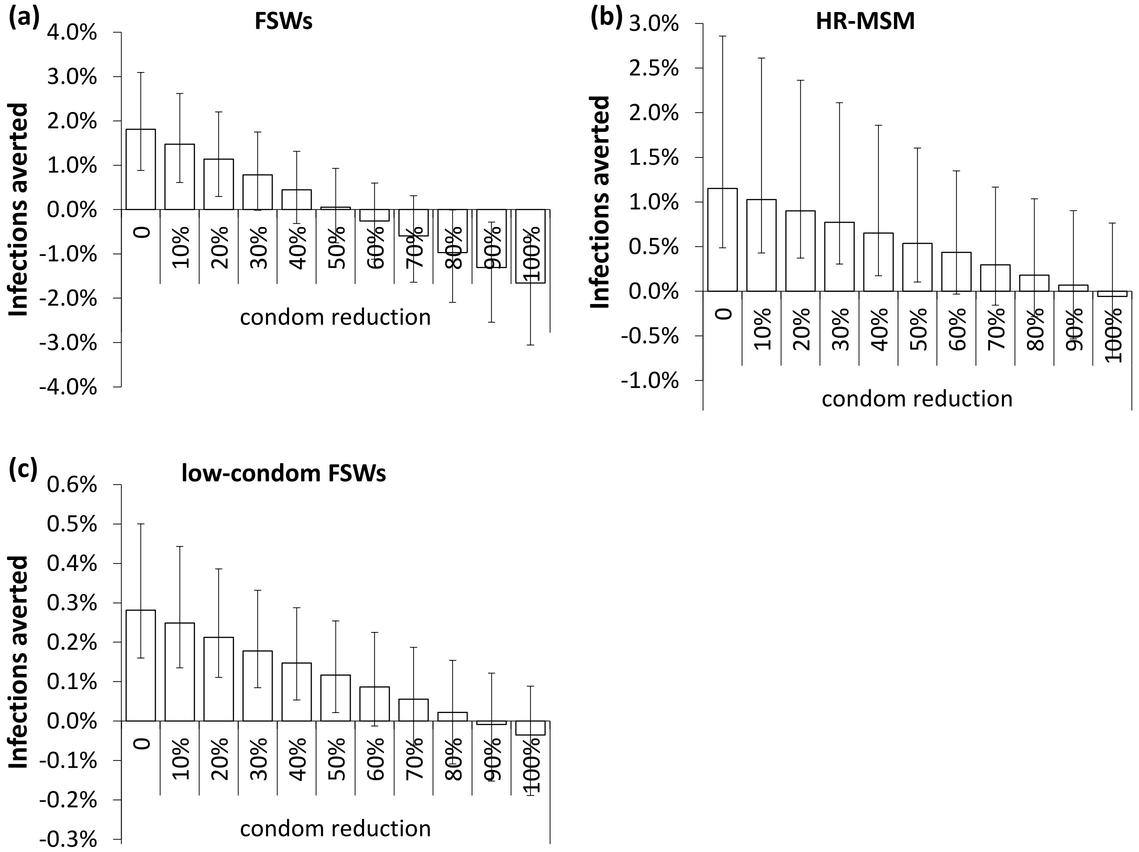

Supplementary table

Table S1: Parameters used in model with sources

Symbol	Parameter	Value	Range	Units	Source
p_0, p_1, p_2, p_3, p_4	Proportion of FSWs and MSM who will never be reached by routine testing	0.05	Fixed	proportion	Assumption – taking into account high testing rates reported by FSW and MSM in IBBA surveys
p_5, p_{12}, p_{13}	Proportion of non-MSM clients and members of the low-risk population who will never be reached by routine testing	0.2	Fixed	proportion	Assumption (consistent with [4])
$\mu_0, \mu_2, \mu_3, \mu_4, \mu_5$	Rate of natural death for current PB, DD, FSW and non-MSM clients	0.021	fixed	Per person per year	Inverse of average remaining life expectancy [5] for mean group age in IBBA data
μ_1	Rate of natural death for current KH	0.019	fixed	Per person per year	Inverse of average remaining life expectancy [5] for mean group age in IBBA data
μ_6	Rate of natural death for former PB	0.032	0.025-0.038	Per person per year	Inverse of remaining life expectancy [5] for PB at cessation of sex with men
μ_7	Rate of natural death for former KH	0.026	0.023-0.029	Per person per year	Inverse of remaining life expectancy [5] for KH at cessation of sex with men
μ_8	Rate of natural death for former DD	0.029	0.025-0.033	Per person per year	Inverse of remaining life expectancy [5] for DD at cessation of sex with men
μ_9, μ_{10}	Rate of natural death for former FSWs	0.025	0.023-0.026	Per person per year	Inverse of remaining life expectancy [5] for FSW at cessation of commercial sex
μ_{11}	Rate of natural death for former non-MSM clients	0.029	0.025-0.033	Per person per year	Inverse of remaining life expectancy [5] for clients at cessation of buying sex

μ_{12}, μ_{13}	Rate of natural death for low-risk population	0.024	fixed	Per person per year	Inverse of average remaining life expectancy [5] for those aged 30
$1/\gamma_1$	Duration of acute HIV stage	0.242	0.103-0.381	years	[6]
$1/\gamma_2$	Time taken to reach CD4 = 500 cells/µl	1.19	1.12-1.26	years	[7]
$1/\gamma_3$	Time taken for CD4 count to drop from 500 to 350 cells/ µl	3	2.9-3.09	years	[7]
$1/\gamma_4$	Time taken for CD4 count to drop from 350 to 200 cells/ µl	3.74	fixed	years	[7]
$1/\gamma_5$	Time from CD4 200 cells/µl to start of pre-AIDS stage	1.37	1.12-1.62	years	[6, 8]
$1/\gamma_6$	Duration of pre-AIDS stage	0.75	0.40-1.10	years	[6]
$1/\gamma_7$	Duration of AIDS stage	0.83	0.61-1.06	years	[6]
χ	Rate of movement of FSWs from low condom to high condom group	Time-varying	-	Per year	At each time step, number moved across to match estimated proportion of FSWs using condoms consistently, calculated from FSW IBBA data [9]
ψ_0	Rate at which PB cease having sex with men	0.064	0.042-0.085	Per person per year	Range covers average current duration up to double this (MSM round 1 IBBA)
ψ_1	Rate at which KH cease having sex with men	0.066	0.044-0.088	Per person per year	Range covers average current duration up to double this (MSM round 1 IBBA)
ψ_2	Rate at which DD cease having sex with men	0.081	0.054-0.108	Per person per year	Range covers average current duration up to double this (MSM round 1 IBBA)
ψ_3, ψ_4	Rate at which FSWs stop selling sex	0.292	0.273-0.312	Per person per year	Estimated from compartmental model fitted to duration data from FSW round 1 IBBA [10]
ψ_5	Rate at which clients stop buying sex	0.098	0.091-0.105	Per person per year	Estimated from compartmental model fitted to duration data from FSW round 1 IBBA [10]

$\tau_{0,Max}, \tau_{1,Max}, \tau_{2,Max}$	% of MSM testing for HIV annually after 2009	58	50-66	%	MSM round 2 IBBA
$\tau_{3,Max}, \tau_{4,Max}$	% of FSW testing for HIV annually after 2011	90	87-92	%	FSW round 3 IBBA
$\tau_{5,Max}, \tau_{6,Max}, \tau_{7,Max}, \tau_{8,Max}, \tau_{9,Max}, \tau_{10,Max}, \tau_{11,Max}, \tau_{12,Max}, \tau_{13,Max}$	% of clients, former MSM, former FSW, low-risk population testing for HIV annually after 2007	11	7-14	%	Client round 1 IBBA extrapolated from 'ever tested' data
Test_slope_HR	Annual absolute increase in % of FSW and MSM tested for HIV	11.5	9.5-13.5	%	Best fit slope to FSW R1, R2 and R3 IBBA data
Test_slope_other	Annual absolute increase in % of clients, former MSM, former FSW, low-risk population tested for HIV	2.6	1.8-3.5	%	Assuming testing increased linearly from 0 in 2003 up to estimated level in 2007
r_0, r_1, r_2	Proportion of MSM offered and initiating PrEP upon testing negative for HIV to achieve required PrEP coverage	20%: $0.105/\tau_{i,Max}$<br>60%: $0.59/\tau_{i,Max}$	fixed	proportion	Calculated to give required PrEP coverage of MSM after 5 years, given testing rate
r_4, r_5	Proportion of FSW offered and initiating PrEP upon testing negative for HIV to achieve required PrEP coverage	20%: 0.17<br>60%: 1.0	fixed	proportion	Calculated to give required PrEP coverage of FSW after 5 years
ν	Rate of PrEP dropout, all groups	0.2	fixed	Per person per year	Assumption
ϵ_{Max}	Proportion linking to pre-ART care, all groups, from 2011 onwards	0.8	0.7-0.9	proportion	From Karnataka linkage data [11]
ω	Ratio of dropout from pre-ART care relative to rate of dropout from ART	2	1-3	ratio	Assumed to be same or greater than rate of dropout from ART
δ	Factor by which HIV progression rates are multiplied when on vs off ART	1/3	fixed		[12]
ϕ	Rate of ART dropout	0.04	0.01-0.07	Per year	Karnataka and India estimates [13, 14]
$a_{1,7}$	Rate of initiating ART due to	1	0-2	Per year	Assumption

	symptoms in AIDS stage				
Ratio $a_{1,6}$: $a_{1,7}$	Relative rate of initiating ART due to symptoms in pre-AIDS stage relative to AIDS stage		0.1-1	Ratio	Assumption
Ratio $a_{1,5}$: $a_{1,6}$	Relative rate of initiating ART due to symptoms with CD4<200 relative to those in pre-AIDS stage		0.1-1	Ratio	Assumption
$a_{2,i}$	Rate at which those in pre-ART care, in HIV stage i, initiate ART	Pre-2004, 0 all i<br><br>2004-2011, 2 if CD4<200, 0 otherwise<br><br>2011 onwards, 2 if CD4<350, 0 otherwise	fixed	Per year	Assumes CD4 testing every 6 months, consistent with [4]. Changes over time reflect changes in national ART guidelines for India.
α	Per-sex-act efficacy of ART in reducing HIV transmission risk in anal or vaginal sex	92	26-100 (triangular distribution used)	%	[15-18]
f_v	Per-sex-act efficacy of condoms in reducing HIV transmission risk in vaginal sex	80	66-94	%	[19, 20]
f_a	Per-sex-act efficacy of condoms in reducing HIV transmission risk in anal sex		61-94	%	[19, 20], accounting for condom breakage reported in MSM round 1 IBBA
κ	Per-sex-act efficacy of PrEP in reducing HIV transmission risk	93	fixed	%	[21, 22]
$e_{c,0}$	Percentage of commercial sex acts and sex acts between MSM in which a condom is used in 1986	5	0-10	%	[9]
β_r	Risk of infection per receptive anal sex act with an infected partner	0.0135	0.002-0.025		[23, 24]

β_{vr}	Risk of HIV infection per receptive vaginal sex act with an infected partner	0.0033	0.0006-0.006		[19, 25, 26]
Cdm_con_red_factor	Factor by which reported condom use by FSWs and MSM is multiplied to take into account over-reporting of condom use		0.79-1		Ratio between FSW- and client-reported condom use at last commercial sex act, IBBA data
$e_{c,1}$(MSM)	Percentage of anal sex acts between MSM in which a condom is used in 2007	70.5	63.7-77.2	%	IBBA round 1 data for MSM
Cdm_slope_FSW	Absolute yearly increase in condom use by FSWs (in commercial sex acts)	4.5	2.1-6.9	%	Estimated from R2 IBBA data; for methods, see [9]
Cdm_slope_MSM	Absolute yearly increase in condom use by MSM (in sex acts with other MSM)	6.0	3.4-8.6	%	Estimated from R1 IBBA data; for methods, see [9]
m_1	Relative infectiousness of those in the acute stage of HIV infection compared with the chronic stage	11.7	4.5-18.8		[25]
m_2	Relative infectiousness of those in the pre-AIDs stage of infection compared with the chronic stage	8.15	4.4-11.9		[25]
x_{KH}	% of kothi/hijra anal sex acts with MSM which are insertive	0.1	0.03-0.17		SBS survey MSM [27]
x_{DD}	% of double decker anal sex acts with MSM which are insertive	0.37	0.28-0.46		SBS survey MSM [27]
x_{PB}	% of panthi/bisexual anal sex acts with MSM which are insertive	0.79	0.7-0.88		SBS survey MSM [27]
κ_a	Relative risk of acquiring HIV from insertive vs. receptive anal sex	0.3	0.1-0.5		[23, 24, 28]
κ_v	Relative risk of acquiring HIV from insertive vs. receptive vaginal sex		0.5-1.0		[19, 25]
MSM_visiting_FSW_DD	% of DD who buy sex from FSWs	13.9	5.8-22.0	%	MSM IBBA R1 data

MSM_visiting_FSW_KH	% of KH who buy sex from FSWs	4.9	1.6-8.2	%	MSM IBBA R1 data
MSM_visiting_FSW_PB	% of PB who buy sex from FSWs	21.4	11.8-31.0	%	MSM IBBA R1 data
Percent_reg_partner_client	% of clients who have a regular female partner	67.0	61.6-72.0	%	Client round 1 IBBA data
Percent_reg_partner_DD	% of DD who have a regular female partner	19.3	10.5-32.7	%	MSM round 1 IBBA data
Percent_reg_partner_FSW	% of FSW who have a regular male partner	69.0	62.8-74.5	%	FSW round 1 IBBA data
Percent_reg_partner_KH	% of KH who have a regular female partner	2.1	0.7-6.1	%	MSM round 1 IBBA data
Percent_reg_partner_PB	% of PB who have a regular female partner	34.7	23.9-47.4	%	MSM round 1 IBBA data
Percent_reg_partner_low_risk_ women	% of low risk women who have a regular male partner	72.4	Fixed	%	National Family Health Survey 3, 2005-2006, Karnataka [29]
popDD	Size of DD population in Bangalore in 2011	9028	2490-155565		[30] [31]
popFSW	Size of FSW population in Bangalore in 2011	12438	9950-14926		[30]
popKH	Size of KH population in Bangalore in 2011	9339	6226-12452		[30] [31]
g1	Annual growth rate of total Bangalore population	3.4	fixed	%	Fitted to data from census surveys 1981-2011
Prop_FSW_sexacts_anal_client	Proportion of commercial sex acts between non-MSM clients and FSW which are anal	4	0-8	%	From client round 1 IBBA, of those not reporting MSM activity, % reporting anal sex with FSWs*proportion of FSWs had anal sex with last 6 months
Prop_FSW_sexacts_anal_DD, Prop_FSW_sexacts_anal_KH, Prop_FSW_sexacts_anal_PB	Proportion of commercial sex acts between MSM and FSW which are anal	8	0-16	%	From client round 1 IBBA, of those reporting MSM activity, % reporting anal sex with FSWs*proportion of FSWs had anal sex with last 6 months
Seedclient, seedDD, seed FSW,	% of each group which is HIV	2	0-4	%	[32]

seedKH, seed PB	positive when infection is seeded in the model in 1986 (each group is seeded independently)				
Sexacts_FSW_DD	For DD who buy sex from FSWs, number of sex acts with FSW per year	28.5	8-49	Per year	MSM IBBA R1
Sexacts_FSW_KH	For KH who buy sex from FSWs, number of sex acts with FSW per year	37.5	8-67	Per year	MSM IBBA R1
Sexacts_FSW_PB	For PB who buy sex from FSWs, number of sex acts with FSW per year	38.5	16-61	Per year	MSM IBBA R1
Sexacts_reg_anal	For those who have a regular partner, number of anal sex acts per year	2.12	0-6.84	Per year	Average and range for values for clients (IBBA R1), FSW (SBS) and MSM (SBS)
Sexacts_reg_vaginal	For those who have a regular partner, number of vaginal sex acts per year	108.4	90.8-127.6	Per year	Average values for clients (IBBA R1), FSW (IBBA R1) and MSM (IBBA R1)
Sexactsclients	Commercial sex acts per year for non-MSM clients	19.6	17.9-21.4	Per year	IBBA R1 client survey, excluding those reporting MSM activity; 12* number of FSWs reported last month
sexactsDD	MSM sex partners per year for DD	69.5	53.0-86.0	Per year	IBBA R2 MSM survey; 12* number of MSM partners reported last month
sexactsFSW	Commercial sex acts per year for FSW	384.0	359.0-410.0	Per year	IBBA R1 FSW survey; 12* number of clients reported last month
sexactsKH	MSM sex partners per year for KH	130.5	93.0-168.0	Per year	IBBA R2 MSM survey; 12* number of MSM partners reported last month
sexactsPB	MSM sex partners per year for PB	49.0	2.0-96.0	Per year	IBBA R2 MSM survey; 12* number of MSM partners reported last month; lower bound lowered to take into account lower risk PB not captured in the survey
CdmCon_reg	% of sex acts in which a condom is	16.1	5.1-27.0	%	Average values for clients (IBBA

	used in regular partnerships				R1), FSW (IBBA R1) and MSM (IBBA R1)
Duration_reg_partnership	Average duration of regular partnerships		9.1-18.2	Years	Average duration of current main partnership for clients (IBBA R1), FSW (IBBA R1) and MSM (IBBA R1), up to double this
CdmConR3_FSW	Percentage of commercial sex acts in which condom used in 2011	90.0	87.3-92.7	%	FSW round 3 IBBA
Cdm_use_always_FSW	% of commercial sex acts in which condoms used by FSWs reporting ‘always’ using condoms with new clients (high-condom FSWs)	99.5	99.1-99.8	%	FSW IBBA
Cdm_use_sometimes_FSW	% of commercial sex acts in which condoms used by FSWs reporting ‘often/sometimes’ using condoms with new clients (part of low-condom FSWs)	77.1	71.5-82.7	%	FSW IBBA
Cdm_use_never_FSW	% of commercial sex acts in which condoms used by FSWs reporting ‘never’ using condoms with new clients (part of low-condom FSWs)	13.0	2.6-23.3	%	FSW IBBA
Cdm_use_always_MSM	% of MSM sex acts in which condoms used by MSM reporting ‘always’ using condoms with partners other than their main partner	96.6	93.9-99.3	%	MSM IBBA
Cdm_use_sometimes_MSM	% of MSM sex acts in which condoms used by MSM reporting ‘sometimes/often’ using condoms with partners other than their main partner	78.1	63.5-92.7	%	MSM IBBA
Cdm_use_never_MSM	% of MSM sex acts in which condoms used by MSM reporting	7.0	0.7-13.2	%	MSM IBBA

	‘never’ using condoms with partners other than their main partner				

IBBA: Integrated behavioural and biological assessments; SBS: special behavioural survey; PB: *panth*i/bisexual MSM groups; DD: double decker MSM group; KH: *kothi*/*hijra* MSM groups

References

1. Blower SM, Dowlatabadi H. Sensitivity and uncertainty analysis of complex models of disease transmission - an HIV model, as an example. *Int Stat Rev* 1994,**62**:229-243.
2. India Health Action Trust. HIV/AIDS situation and response in Bangalore urban district: epidemiological appraisal using data triangulation. Bangalore, India: India Health Action Trust; 2010.
3. Banandur P, Rajaram SP, Mahagaonkar SB, Bradley J, Ramesh BM, Washington RG, *et al.* Heterogeneity of the HIV epidemic in the general population of Karnataka state, south India. *BMC Public Health* 2011,**11 Suppl 6**:S13.
4. Eaton JW, Menzies NA, Stover J, Cambiano V, Chindelevitch L, Cori A, *et al.* Health benefits, costs, and cost-effectiveness of earlier eligibility for adult antiretroviral therapy and expanded treatment coverage: a combined analysis of 12 mathematical models. *Lancet Glob Health* 2014,**2**:e23-34.
5. WHO. WHO global health observatory data repository. Life expectancy: life tables by country. 2012. http://apps.who.int/gho/data/node.main.LIFECOUNTRY?lang=en [Accessed 01/09/2016]
6. Hollingsworth TD, Anderson RM, Fraser C. HIV-1 transmission, by stage of infection. *J Infect Dis* 2008,**198**:687-693.
7. Lodi S, Phillips A, Touloumi G, Geskus R, Meyer L, Thiebaut R, *et al.* Time from human immunodeficiency virus seroconversion to reaching CD4+ cell count thresholds <200, <350, and <500 cells/mm^3: assessment of need following changes in treatment guidelines. *Clin Infect Dis* 2011,**53**:817-825.
8. eART-linc collaboration, Wandel S, Egger M, Rangsin R, Nelson KE, Costello C, *et al.* Duration from seroconversion to eligibility for antiretroviral therapy and from ART eligibility to death in adult HIV-infected patients from low and middle-income countries: collaborative analysis of prospective studies. *Sex Transm Infect* 2008,**84(Suppl 1)**:i31-i36.
9. Lowndes CM, Alary M, Verma S, Demers E, Bradley J, Jayachandran AA, *et al.* Assessment of intervention outcome in the absence of baseline data: 'reconstruction' of condom use time trends using retrospective analysis of survey data. *Sex Transm Infect* 2010,**86**:I49-I55.
10. Pickles M, Boily M-C, Vickerman P, Lowndes CM, Moses S, Blanchard JF, *et al.* Assessment of the population-level effectiveness of the Avahan HIV-prevention programme in South India: a preplanned, causal-pathway-based modelling analysis. *The Lancet Global Health* 2013,**1**:e289-299.
11. National AIDS Control Organisation. ART scale up in Karnataka. Bangalore: NACO; 2011.
12. Johansson KA, Robberstad B, Norheim OF. Further benefits by early start of HIV treatment in low income countries: survival estimates of early versus deferred antiretroviral therapy. *AIDS Res Ther* 2010,**7**:3.
13. Karnataka State AIDS Prevention Society. Annual Action Plan 2012-2013. Bangalore: Karnataka State AIDS Prevention Society; 2012.
14. UNAIDS. UNGASS country progress report India. New Delhi: National AIDS Control Organisation; 2010.
15. Attia S, Egger M, Müller M, Zwahlen M, Low N. Sexual transmission of HIV according to viral load and antiretroviral therapy: systematic review and meta-analysis. *AIDS* 2009,**23**:1397-1404.

16. Donnell D, Baeten JM, Kiarie J, Thomas KK, Stevens W, Cohen CR, *et al.* Heterosexual HIV-1 transmission after initiation of antiretroviral therapy: a prospective cohort analysis. *Lancet* 2010,**375**:2092-2098.
17. Jia Z, Ruan Y, Li Q, Xie P, Li P, Wang X, *et al.* Antiretroviral therapy to prevent HIV transmission in serodiscordant couples in China (2003-11): a national observational cohort study. *Lancet* 2013,**382**:1195-1203.
18. Cohen MS, Chen YQ, McCauley M, Gamble T, Hosseinipour MC, Kumarasamy N, *et al.* Prevention of HIV-1 infection with early antiretroviral therapy. *N Engl J Med* 2011,**365**:493-505.
19. Hughes JP, Baeten JM, Lingappa JR, Magaret AS, Wald A, de Bruyn G, *et al.* Determinants of per-coital-act HIV-1 infectivity among African HIV-1-serodiscordant couples. *J Infect Dis* 2012,**205**:358-365.
20. Weller SC, Davis-Beaty K. Condom effectiveness in reducing heterosexual HIV transmission. *Cochrane Database Syst Rev* 2002:CD003255.
21. Grant RM, Lama JR, Anderson PL, McMahan V, Liu AY, Vargas L, *et al.* Preexposure chemoprophylaxis for HIV prevention in men who have sex with men. *N Engl J Med* 2010,**363**:2587-2599.
22. Donnell D, Baeten JM, Bumpus NN, Brantley J, Bangsberg DR, Haberer JE, *et al.* HIV protective efficacy and correlates of tenofovir blood concentrations in a clinical trial of PrEP for HIV prevention. *J Acquir Immune Defic Syndr* 2014,**66**:340-348.
23. Baggaley RF, White RG, Boily MC. HIV transmission risk through anal intercourse: systematic review, meta-analysis and implications for HIV prevention. *Int J Epidemiol* 2010,**39**:1048-1063.
24. Jin FY, Jansson J, Law M, Prestage GP, Zablotska I, Imrie JCG, *et al.* Per-contact probability of HIV transmission in homosexual men in Sydney in the era of HAART. *AIDS* 2010,**24**:907-913.
25. Boily MC, Baggaley RF, Wang L, Masse B, White RG, Hayes RJ, *et al.* Heterosexual risk of HIV-1 infection per sexual act: systematic review and meta-analysis of observational studies. *Lancet Infect Dis* 2009,**9**:118-129.
26. Mahiane SG, Legeai C, Taljaard D, Latouche A, Puren A, Peillon A, *et al.* Transmission probabilities of HIV and herpes simplex virus type 2, effect of male circumcision and interaction: a longitudinal study in a township of South Africa. *AIDS* 2009,**23**:377-383.
27. Phillips AE, Boily MC, Lowndes CM, Garnett GP, Gurav K, Ramesh BM, *et al.* Sexual identity and its contribution to MSM risk behavior in Bangaluru (Bangalore), India: the results of a two-stage cluster sampling survey. *J LGBT Health Res* 2008,**4**:111-126.
28. Vittinghoff E, Douglas J, Judson F, McKirnan D, MacQueen K, Buchbinder SP. Per-contact risk of human immunodeficiency virus transmission between male sexual partners. *Am J Epidemiol* 1999,**150**:306-311.
29. International Institute for Population Science (IIPS) and Macro International. National Family Health Survey (NFHS-3), India, 2005-2006: Karnataka. Mumbai: IIPS; 2008.
30. Bill & Melinda Gates Foundation. Use it or lose it: How Avahan used data to shape its HIV prevention efforts in India. New Delhi: BMGF; 2008.
31. Lowndes CM, Jayachandran AA, Banandur P, Ramesh BM, Washington R, Sangameshwar BM, *et al.* Polling booth surveys: a novel approach for reducing social desirability bias in HIV-related behavioural surveys in resource-poor settings. *AIDS Behav* 2012,**16**:1054-1062.

32. Simoes EAF, Babu PG, Jeyakumari HM, John TJ. The initial detection of human immunodeficiency virus-1 and its subsequent spread in prostitutes in Tamil Nadu, India. *J Acquir Immune Defic Syndr Hum Retrovirol* 1993,**6**:1030-1034.
